# Niche differentiation in microbial communities with stable genomic traits over time in engineered systems

**DOI:** 10.1093/ismejo/wrae042

**Published:** 2024-03-12

**Authors:** Jinjin Yu, Justin Y Y Lee, Siang Nee Tang, Patrick K H Lee

**Affiliations:** School of Energy and Environment, City University of Hong Kong, Kowloon, Hong Kong SAR, China; School of Energy and Environment, City University of Hong Kong, Kowloon, Hong Kong SAR, China; Facility Management and Environmental Engineering, TAL Group, Kowloon, Hong Kong SAR, China; School of Energy and Environment and State Key Laboratory of Marine Pollution, City University of Hong Kong, Kowloon, Hong Kong SAR, China

**Keywords:** genome-resolved metagenomics, plant-specific indicators, niche differentiation, genomic traits, microbial succession, process kinetics

## Abstract

Microbial communities in full-scale engineered systems undergo dynamic compositional changes. However, mechanisms governing assembly of such microbes and succession of their functioning and genomic traits under various environmental conditions are unclear. In this study, we used the activated sludge and anaerobic treatment systems of four full-scale industrial wastewater treatment plants as models to investigate the niches of microbes in communities and the temporal succession patterns of community compositions. High-quality representative metagenome-assembled genomes revealed that taxonomic, functional, and trait-based compositions were strongly shaped by environmental selection, with replacement processes primarily driving variations in taxonomic and functional compositions. Plant-specific indicators were associated with system environmental conditions and exhibited strong determinism and trajectory directionality over time. The partitioning of microbes in a co-abundance network according to groups of plant-specific indicators, together with significant between-group differences in genomic traits, indicated the occurrence of niche differentiation. The indicators of the treatment plant with rich nutrient input and high substrate removal efficiency exhibited a faster predicted growth rate, lower guanine–cytosine content, smaller genome size, and higher codon usage bias than the indicators of the other plants. In individual plants, taxonomic composition displayed a more rapid temporal succession than functional and trait-based compositions. The succession of taxonomic, functional, and trait-based compositions was correlated with the kinetics of treatment processes in the activated sludge systems. This study provides insights into ecological niches of microbes in engineered systems and succession patterns of their functions and traits, which will aid microbial community management to improve treatment performance.

## Introduction

Taxonomically diverse microbes, with extensive metabolic functions, occupy distinct ecological niches in various ecosystems [1], such as the human gut [2], soil [3], aquatic environments [4], and engineered bioreactors [5]. Due to environmental selection, the spatiotemporal distributions of microbes are strongly governed in an environment-dependent manner [6, 7]. Microbes in a given ecosystem exhibit individual responses to environmental changes, as they occupy different ecological niches [8, 9]. This generates divergent dynamics between community members, such as abundant taxa and rare taxa. Rare taxa typically occupy narrower ecological niches and experience stronger environmental filtering than abundant taxa, but rare taxa can also help to maintain ecosystem homeostasis [10, 11]. Therefore, the temporal dynamics and interactions of microbes in a community, and the ecological mechanisms of their succession, must be determined to reveal ecosystem functioning [12, 13].

Profiling the spatiotemporal dynamics of microbial populations based on phylogeny partly reveals the mechanisms of microbial succession [14]. However, genomic traits are linked to the fitness and performance (e.g., metabolism and growth rate) of microbes in response to environmental conditions [15], and thus to ecosystem structure and functioning [16]. Moreover, genomic traits such as genome size [17], guanine–cytosine (GC) content [18], rRNA gene copy number [19], growth rate [20], and codon usage bias [21] reflect optimized life-history strategies, i.e. how microbes utilize resources [22]. Thus, trait-based analysis at the species and community levels is an alternative, phylogeny-independent approach for assessing the ecological niches of microbes in a community and predicting their ecosystem functioning [23].

The temporal succession of microbial communities reflects assembly mechanisms [24] and occurs in a divergent, convergent, idiosyncratic, linear, or lagged manner [13, 25]. Moreover, the temporal dynamics of communities is needed to predict the responses of biodiversity and ecosystem functioning to temporal environmental changes [26, 27]. The succession of community populations is linked not only to differences between microbes’ growth, life-history strategies, and longevity but also to the stability and resilience of microbial systems [28]. Thus far, most studies on succession have predominantly focused on taxonomic compositions [29, 30]; only a few studies have explored the dynamics of community functions and traits and their correlations with environmental conditions [9, 23, 31].

Biological wastewater treatment plants (WWTPs) harbor diverse microbial assemblages that are strongly influenced by operating conditions [5, 32]. Thus, WWTPs are ideal models for investigating the dynamics of microbes in communities and their succession patterns under fluctuating environmental conditions in engineered systems [33]. Accordingly, we used four full-scale WWTPs processing wastewater from similar sources as models and employed reconstructed high-quality (HQ) representative metagenome-assembled genomes (rMAGs) to gain insights into niche differentiation between microbes within communities and temporal succession patterns in their functions and genomic traits. We aimed to determine the (1) ecological niches of microbes in communities and their assembly mechanisms, (2) species- and community-level genomic traits of microbial communities, and (3) partitioning and succession patterns in taxonomic, functional, and trait-based compositions and their potential correlations with environmental conditions in WWTPs. We speculated that microbes in communities would exhibit divergent assembly patterns and genomic traits due to differences between their ecological niches, and that microbial functions and genomic traits would exhibit greater stability over time than microbial taxonomy.

## Materials and methods

### W‌WTPs and chemical oxygen demand removal kinetics

Four full-scale WWTPs (denoted “IG,” “TG,” “TV,” and “VNG”) located in Southeast Asia, each processing wastewater from a different textile mill, were used as model systems. During the sampling period, all of the mills produced similar textile products. In addition, plant TG processed mixed municipal and textile wastewater, whereas the others processed only textile wastewater. Plant VNG had an additional set of upflow anaerobic sludge blanket, anaerobic treatment (AT) tanks, and activated sludge (AS) tanks (Fig. S1A). The treatment processes were configured as continuous stirred tank reactors, with working volumes ranging from 147 to 752 m^3^ for AS tanks and from 79 to 576 m^3^ for AT tanks, and synthetic cotton carriers were anchored to the bottom of some of the tanks to retain biomass.

Wastewater samples were collected from the influent and inside of each individual AS and AT tank to measure and calculate 13 environmental parameters, such as the chemical oxygen demand (COD) concentration and COD removal efficiency, using standard methods [34]. The COD removal efficiency and rate of each individual system were calculated by subtracting the COD concentrations in the influent wastewater from those in the effluent wastewater of the corresponding system. The environmental conditions of the systems were described using all 13 parameters, the details of which have been reported previously [35], and the key information is summarized in Figs S2 and S3. The measured and calculated data were normalized using the “min–max normalization” method with the “scale()” function in R (v4.1.2). Between-plant differences in the environmental conditions of the respective AS and AT systems were calculated based on Euclidean distance using the vegan package (v2.5.6) in R.

First-order [36] and Grau second-order [37] models were fitted to the combined temporal COD data of all the AS systems. A linear model with the plant as the additional fixed effect was used to evaluate the fit of the two kinetic models, and that yielding a higher coefficient of determination (*R*^2^) was adopted to estimate the COD removal kinetics in the AS system of each plant. Kinetic modeling was not performed for the AT systems, as they only performed preliminary processes. Kinetic coefficient estimation is detailed in Text S1 in the Supporting Information (SI).

### Sampling and metagenomic sequencing

From October 2018 to October 2019, samples were collected from the AS and AT tanks of the four plants (Fig. S1A). Planktonic wastewater samples were collected from inside each individual tank biweekly by filtration, and biofilm samples were obtained monthly by scraping materials from cotton carriers. Genomic DNA was extracted using the DNeasy PowerSoil Kit (Qiagen, Germantown, MD).

Metagenomic sequencing of 146 AS, 186 AT samples, and three negative controls was performed on a NovaSeq platform (Illumina) to generate 150-bp paired-end reads (Table S1). An average of ~14.8 million raw paired-end reads were generated per AS or AT sample. The raw metagenomic reads were processed to give an average of ~14.3 million HQ paired-end reads per sample. As the negative controls yielded only ~58 000 paired-end reads per sample, no further decontamination procedures were performed for the AS and AT samples. Sampling and sequencing are detailed in Text S1 in the SI. The coverage of AS and AT samples was estimated using Nonpareil [38] (v.3.401) with parameters-T kmer -X 1000. Only the forward reads of each sample were used to avoid dependency of the paired reads, which can bias the estimates. Estimation of the sequencing coverage showed that sequencing captured averages of 68.1 ± 6.6% and 73.6 ± 6.2% of the microbial communities in the AS and AT samples, respectively.

### Microbial community composition based on short reads and reconstruction of MAGs

HQ metagenomic short-reads were analyzed using SingleM (v0.13.2, https://github.com/wwood/singlem) to profile taxonomic compositions. MEGAHIT [39] (v1.2.9) was used to assemble the HQ reads of each sample into contigs, and only contigs longer than 1000 bp were retained. The retained contigs in each sample were binned into MAGs using MaxBin2 [40] (v2.2.7). The completeness and contamination of the MAGs were assessed using CheckM [41] (v1.1.3) with default parameters. Only MAGs of high (completeness >90% and contamination <5%) and medium quality (50% < completeness <90% and contamination <5%) were retained for downstream analyses [42]. Dereplication of MAGs from the respective AS and AT systems of all four plants was performed using dRep [43] (v2.6.2) with clustering thresholds of 90% Mash similarity for the primary clustering and 99% average nucleotide identity for the secondary clustering.

### Taxonomic and functional annotation of rMAGs

The taxonomy of the medium- and HQ rMAGs after dereplication was assigned according to the R06-RS202 database using GTDB-Tk [44] (v1.5.0). A phylogenetic tree of the rMAGs was constructed using GTDB-Tk with the default settings and visualized using iTOL [45] (v6). CoverM (v0.5.0, https://github.com/wwood/CoverM) was used to determine the read coverage of the rMAGs, and those that passed the 10% coverage threshold (—min-covered-fraction 10) were considered to be present in a sample [46]. Additionally, rnammer [47] (v1.2) and tRNAscan-SE [48] (v2.0.7) were employed to identify the presence of 5S, 16S, and 23S rRNAs and tRNA genes in the rMAGs.

The coding sequences (CDSs) of the HQ rMAGs were annotated using Prokka [49] (v1.14.6) with an E-value threshold set at 1 × 10^−6^. All of the CDSs identified in the HQ rMAGs were annotated based on the Kyoto Encyclopedia of Genes and Genomes (KEGG) functional orthologs (KOs) using EnrichM (v0.5.0, https://github.com/geronimp/enrichM). The annotated functions were used to determine the presence or absence of a specific KEGG module in which only pathways where all the genes were present were considered to be complete in a genome. The METABOLIC-G function of METABOLIC [50] (v4.0) with the threshold set to “-m-cutoff 0.8” was used to predict pathways related to carbon and nitrogen metabolisms in the HQ rMAGs. The relative abundance of KOs or pathways in a sample was defined as the sum of the copy number of KOs in all the HQ rMAGs multiplied by the abundance (i.e. read coverage) of the corresponding HQ rMAGs.

### Microbial community diversity

The α-diversity of microbial communities was assessed using the R package vegan (v2.5.6) and based on the count results of the 14 single-copy marker genes obtained from SingleM. The β-diversity of the taxonomic and functional compositions based on the HQ rMAGs was estimated by calculating the Bray–Curtis dissimilarity. Compositional differences between the plants (IG, TG, TV, and VNG) or sample types (planktonic and biofilm) were assessed using permutational multivariate analysis of variance (PERMANOVA) and visualized using principal component analysis. The relationship between taxonomic and functional compositions based on the HQ rMAGs was determined by performing a Mantel test with 999 permutations using the R package vegan (v2.5.6). The correlation between the taxonomic and functional dissimilarities was determined based on the Pearson correlation coefficient (*r*), with *r* = 1 and *r* = 0 indicating that a microbial community was strictly functionally dependent and redundant, respectively [51]. β-diversity was partitioned into replacement processes and richness difference processes using the R package adespatial (v0.3–16), in which the Jaccard dissimilarity (Podani family) was applied. Replacement processes describe the turnover of taxa identities or functions alone, whereas richness difference processes describe the variation in taxonomic or functional richness between each community [52]. The statistical significance of a comparison between two groups (Mann–Whitney test) or more than two groups (Kruskal–Wallis test) was calculated using the R package stats (v4.1.2). *P* < .05 was considered to indicate statistical significance.

### Identification of plant-specific indicators based on the relative abundance of HQ rMAGs

The HQ rMAGs that were indicators of specific plants were identified using correlation-based indicator analyses, in which the point-biserial correlation coefficient was calculated using the R package indicspecies (v1.7.12), with the “r.g” function and 999 permutations based on the relative abundance of HQ rMAGs. The Sloan neutral model [53] was used to determine the assembly mechanisms of plant-specific indicators and non-indicators over time. The normalized stochasticity ratios (NSTs) [54] of the microbial communities were quantified using the R package NST (v3.0.6). Trajectory directionality analysis of microbial community members was performed using the R package ecotraj (v0.1.0). The details of community assembly mechanism and trajectory directionality analyses are described in Text S1 in the SI.

The co-abundance network of all the HQ rMAGs based on relative abundance was constructed using BAnOCC, which is a Bayesian model for detecting significant pairwise associations in compositional data [55]. BAnOCC was executed with 5000 iterations and 1000 warm-up cycles to read convergence in the R package banocc (v1.24.0). A 99% credible interval for the width of the returned credible intervals was used, and only statistically significant and strong correlations with an estimated coefficient of >70% were retained. The co-abundance network was visualized using Gephi software [56] (v0.9.2). In the association network, the module participation of each HQ rMAG was calculated as the proportion of total edges connected to an HQ rMAG with respect to the total number of HQ rMAGs in a specific module [57].

### Genomic traits and trait-based compositions of plant-specific indicators

A set of genomic traits—genome size, GC content, predicted growth rate, and codon usage bias—was determined for HQ rMAGs. The predicted growth rates (i.e. minimum generation times) of the HQ rMAGs were estimated using Growthpred [58] (v1.07) with the parameter “-m -b -c 0 -r -T 30 -S,” and the ribosomal gene codon-usage bias was calculated using the codon adaptation index [59].

Genomic traits were calculated at the community level in individual samples using the community-weighted means (CWMs) method [23] based only on plant-specific indicators to enhance representativeness of the genomic traits of individual plants. Between-sample dissimilarity in trait-based composition was calculated based on the CWMs of the four genomic traits of plant-specific indicators or all HQ rMAGs using the Bray–Curtis dissimilarity. Similarities between trait-based composition and taxonomic or functional composition were determined using the Procrustes and Mantel tests. The CWMs of genomic traits and estimations of trait-based dissimilarity are detailed in Text S1 in the SI. Furthermore, the temporal succession of taxonomic, functional, and trait-based compositions was evaluated by determining the time-decay slope [60] and halving-time [61]. A steep time-decay slope or a short halving-time indicates rapid succession in a community over time. The details of succession estimation using the time-decay slope and halving-time are described in Text S1 in the SI.

## Results

### Environmental conditions and treatment performance differed between plants

The environmental conditions of the AS and AT systems in the four plants have been detailed previously [35] and are summarized in Figs S2 and S3. In brief, the four textile WWTPs were designed and operated mainly for COD removal, and they process an average daily wastewater flow of 208.2 ± 113.3 m^3^. The average hydraulic retention times of the AS and AT systems were 1.5 ± 0.8 and 1.0 ± 0.4 days, respectively. The average COD loadings for the IG, TV, and VNG plants were between 714.0 ± 146.8 and 750.2 ± 149.3 mg/m^3^/d, whereas plant TG had a substantially lower average COD loading of 270.4 ± 79.7 mg/m^3^/d. The COD removal efficiency differed between the four plants in the AS and AT systems (Kruskal–Wallis test, both *P* < .001), with plant TG processing mixed wastewater having the highest average removal efficiency (65.5 ± 10.7% for the AS system and 39.9 ± 10.8% for the AT system). The average total nitrogen concentrations in the four WWTPs were 90 ± 24.3 mg/l in the AT systems and 99.9 ± 28.2 mg/l in the AS systems. Based on the similar measured total nitrogen concentrations observed throughout the treatment train of the four plants, it was determined that the nitrogen removal was limited. The highest ammonium concentration and lowest carbon-to-nitrogen ratio were measured in plant TG. The environmental conditions of the time-series samples were clustered by plant (PERMANOVA, *P* = .001) (Fig. S1B). The first-order model (adjusted-*R*^2^ = 0.68) could better fit the COD data of AS systems than the Grau second-order model (adjusted-*R*^2^ = 0.49), so the former model was adopted to estimate the COD removal kinetics in individual plants. Consistent with the removal efficiency, the COD removal kinetics of the AS system of plant TG was the highest (*K*_1_ = 0.97 ± 0.06 d^−1^) (Table S2), suggesting that this plant outperformed the others.

### Plant-dependent taxonomic compositions of microbial communities

The taxonomic composition of the AS and AT systems was assessed by short-read-based analysis of single-copy ribosomal protein genes. At the phylum level, analysis of the relative abundance revealed that the dominant phyla were *Proteobacteria* (41.7 ± 7.4%) and *Bacteroidota* (7.9 ± 5.2%) in the AS systems and *Proteobacteria* (42.3 ± 12.7%) and *Chloroflexi* (10.1 ± 6.8%) in the AT systems (Fig. S4A). The α-diversity (assessed based on richness, evenness, and the Shannon index) was significantly different between plants for both systems (Kruskal–Wallis test, all *P* < .001) (Fig. S5). The taxonomic composition clustered more strongly by plant (PERMANOVA, both *P* = .001, pseudo-*F* = 25.67 and 33.37 for the AS and AT systems, respectively) than by sample type (planktonic and biofilm; PERMANOVA, both *P* = .001, pseudo-*F* = 3.16 and 6.62 for the AS and AT systems, respectively) (Fig. S4B). Despite variations in the environmental conditions across the AT tanks of plant VNG (Fig. S3), the clustering of the microbial community was observed in all the tanks (Fig. S4B). Therefore, all the samples from multiple anaerobic tanks were combined to represent the AT system of plant VNG for the subsequent microbial community analysis. In both the AS and AT systems across all plants, all environment parameters were significantly associated with short-read-based community variations (PERMANOVA, all *P* < .05), although the contribution of each parameter varied (Fig. S1C). In the AS systems, the COD concentration and its related parameters (e.g. COD removal efficiency and COD removal rate) were identified as the major contributors to variations in community composition (PERMANOVA, R^2^ = 0.08 to 0.10, all *P* = .001). However, in the AT systems, pH (PERMANOVA, R^2^ = 0.09, *P* = .001) and ammonium concentration (PERMANOVA, R^2^ = 0.08, *P* = .001) were identified as the major influencing factors.

### Taxonomic and functional annotation of the reconstructed MAGs

**Figure 1:**
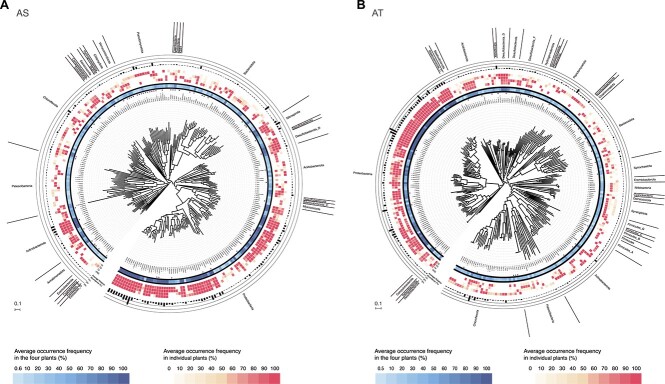
Phylogenetic tree of the medium- and HQ rMAGs in bacterial lineages from the (A) AS and (B) AT systems; the innermost ring shows the lowest possible taxonomic rank of the rMAGs (278 from AS and 282 from AT) based on GTDB-Tk at the genus (prefix “g”) or family (prefix “f”) level, and the outermost ring shows the phylum rank; HQ rMAGs containing all of the 5S, 16S, and 23S rRNA genes and having more than 18 tRNAs are highlighted with a black circle in the innermost ring; scale bar indicates the tree scale; the average occurrence frequencies (in individual plants and the four plants) and average relative abundance in the four plants for the rMAGs over time are indicated by the heatmap and bar chart in the outer rings, respectively.

Sample-specific MAG reconstruction yielded 7694 and 8903 MAGs before dereplication for the AS and AT systems, respectively (Fig. S6A). Examination of these reconstructed MAGs in AS and AT systems showed that they contained 1033 and 1178 medium- and HQ MAGs, respectively. The genome size ranges of these medium- and HQ MAGs were 0.4–10.2 Mb in the AS systems and 0.5–8.7 Mb in the AT systems (Fig. S6B). After dereplication, a subset of 284 and 297 medium- and HQ rMAGs remained in the AS and AT systems, respectively, 26 and 16 of which were present in all of the AS and AT systems, respectively (Fig. 1 and Fig. S7). In total, 57 (from AS systems) and 48 (from AT systems) HQ rMAGs were identified that had all 5S, 16S, and 23S rRNA genes and more than 18 tRNAs (Fig. 1, Figures S6C and S7).

The 284 medium- and HQ rMAGs identified in the AS systems were from 30 phyla, including three archaeal phyla (Fig. 1A and Fig. S7A). Approximately half of the rMAGs in the AS systems were in the phyla *Proteobacteria* (86), *Bacteroidota* (41), and *Chloroflexota* (29), whereas most of the rMAGs in the AT systems were in the phyla *Proteobacteria* (86) and *Chloroflexota* (31), and eight rMAGs were in the archaeal phylum *Halobacteriota* (Fig. 1B and Fig.S7B). Furthermore, 88 and 84 rMAGs from the AS and AT systems, respectively, could not be classified at the genus level and were thus regarded as novel (Fig. S6D).

Only the HQ rMAGs (145 in the AS systems [Table S3] and 169 in the AT systems [Table S4]) were included in subsequent analyses, thereby ensuring that functional annotation of genes was not influenced by genome completeness. The four plants had an average of 75 ± 8 and 85 ± 10 HQ rMAGs in the AS and AT systems, respectively. The HQ rMAGs recruited an average of 49.6 ± 11.5% and 64.8 ± 14.4% of the reads per sample in all four plants in the AS and AT systems, respectively, indicating that the HQ rMAGs were the abundant taxa of the microbial communities in both systems. KEGG module analysis revealed the full set of genes encoding the carbon, nitrogen, and phosphorus pathways in the HQ rMAGs from both systems (Figs S8 and S9). However, genes for acetoclastic and hydrogenotrophic methanogenesis were found only in the HQ rMAGs in the AT systems. Moreover, HQ rMAGs belonging to *Proteobacteria* and *Actinobacteriota* (12 and 10 in the AS and AT systems, respectively) contained genes for aromatic-compound degradation.

### Taxonomic and functional compositions were coupled and driven by replacement processes

Temporal variations in the taxonomic compositions of the microbial communities in the four plants were characterized based on the relative abundance of HQ rMAGs. Taxonomic analysis revealed that over time, members of the phyla *Proteobacteria* (28.5 ± 10.7%), *Planctomycetota* (4.4 ± 4.1%), and *Bacteroidota* (3.7 ± 2.7%) were predominant in the AS systems, whereas members of the phyla *Proteobacteria* (36.8 ± 18.5%), *Halobacteriota* (6.6 ± 15.0%), and *Chloroflexota* (5.9 ± 6.2%) were predominant in the AT systems (Fig. S4A). The taxonomic affiliation of the top abundant taxa identified using HQ rMAGs was comparable to that of the metagenomic short reads, albeit with minor variations in the relative abundance of some taxa. Furthermore, consistent with the metagenomic short reads, the taxonomic compositions based on the relative abundance of HQ rMAGs clustered by plant for both the AS and AT systems (PERMANOVA, both *P* = .001) (Fig. S4B), and the environmental parameters of COD removal efficiency and ammonium concentration were identified as the major contributors to the community variations in the respective AS and AT systems (Fig. S1C). Variations in the functional compositions of the AS and AT microbial communities were assessed based on the relative abundance of KOs in the HQ rMAGs. Consistent with the taxonomic compositions, the functional compositions for both the AS and AT systems clustered by plant (PERMANOVA, both *P* = .001) (Fig. S4B). The between-plant difference in taxonomic composition (pseudo-*F* = 21.43 and 22.85 for the AS and AT systems, respectively) was greater than that in functional composition (pseudo-*F* = 16.61 and 15.05 for the AS and AT systems, respectively), suggesting that the functional compositions of the plants tended to remain more similar than their taxonomic compositions over time.

**Figure 2:**
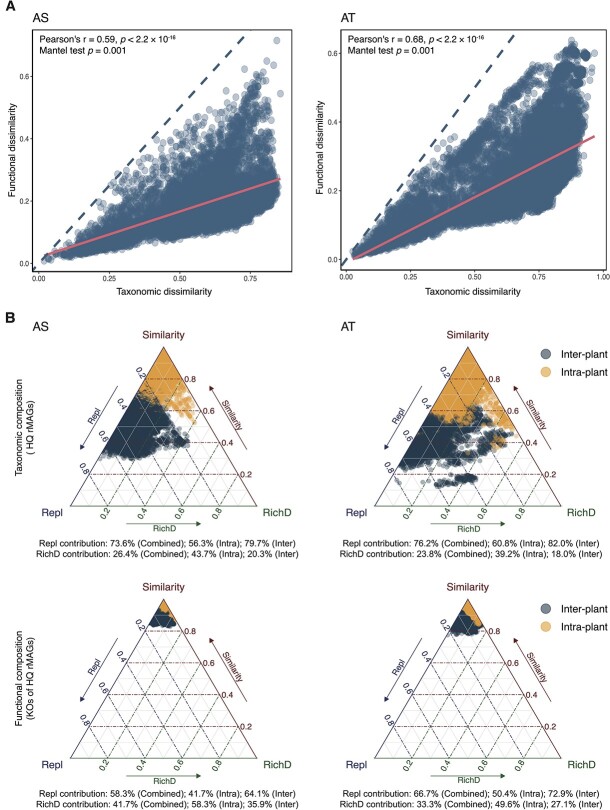
Functional dependency and partitioning of taxonomic and functional composition variations of the HQ rMAGs in the AS and AT systems over time; (A) in the functional dependency analysis, the functional and taxonomic dissimilarities between plants over time were calculated using the Bray–Curtis dissimilarity based on the HQ rMAGs; the solid line represents the linear regression between the taxonomic and functional dissimilarities, and the dashed line represents the strictly dependent 1:1 relationship; some of the data points in the figure may overlap with each other; (B) partitioning of the taxonomic and functional composition dissimilarities of the respective AS and AT systems over time was calculated using the Jaccard dissimilarity (Podani family); each point represents a pair of comparisons between samples over time, and the color indicates the type of comparison (intra- and inter-plant comparisons); the position of each point is determined by a triplet of values from the similarity, replacement (Repl; referring to species or functions turnover), and richness difference (RichD; referring to species or functions gain/loss) matrices, where each triplet sums to 1; the average contributions of replacement and richness difference processes to the temporal compositional dissimilarities in all plants combined (denoted as “combined”), in individual plants (denoted as “intra”), and between plants (denoted as “inter”) are shown; some of the data points in the figure may overlap with each other.

Compared with the functional compositions, the taxonomic compositions consistently yielded higher dissimilarity in both the AS and AT systems (Fig. 2A). The similarities between the taxonomic and functional compositions of the AS and AT systems, respectively, in the four plants over time were assessed using the Mantel test. This revealed a significant and positive correlation (both *P* = .001; Pearson’s *r* = 0.59 and 0.68 for the AS and AT systems, respectively) between the two compositions (Fig. 2A), suggesting that microbial functions were coupled with the taxonomic composition across plants during temporal succession. Partitioning of the variations in taxonomic and functional compositions for the AS and AT systems, respectively, over time in all of the plants showed that both variations were mainly due to replacement processes (i.e. accounted for an average of 73.6% [AS] and 76.2% [AT] of the taxonomic variations, and 58.3% [AS] and 66.7% [AT] of the functional variations). In comparison, richness difference processes contributed less to these variations (i.e. accounted for an average of 26.4% [AS] and 23.8% [AT] of the taxonomic variations, and 41.7% [AS] and 33.3% [AT] of the functional variations) (Fig. 2B). Partitioning of the variations into intra- and inter-plant dissimilarities for the respective AS and AT systems also revealed that both taxonomic and functional compositions were governed predominantly by replacement processes over time (Fig. 2B).

### Niche differentiation and distinct genomic traits of microbial community members

**Figure 3:**
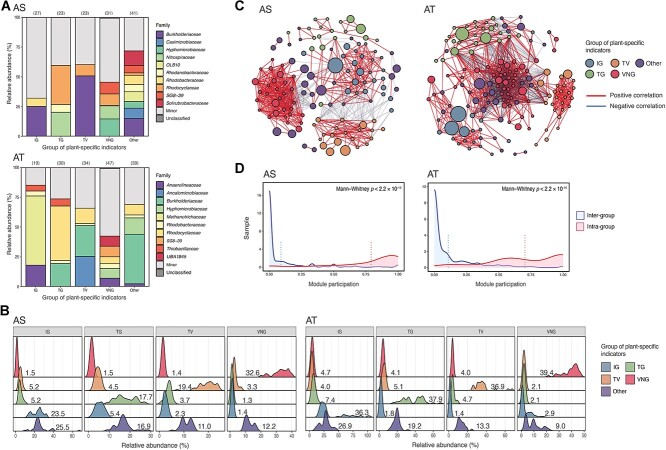
Plant-specific indicators in the AS and AT systems over time; (A) Taxonomic compositions of the groups of plant-specific indicators in the AS and AT systems over time; the “other” group refers to non-indicators; only the top-10 families are shown; other families are grouped under “minor”; the number of HQ rMAGs in each group of plant-specific indicators is shown in brackets at the top; (B) ridge plots showing the relative abundances of each group of plant-specific indicators in different plants during temporal succession; the numbers indicate the average relative abundances; (C) co-abundance network of all HQ rMAGs in the respective AS and AT systems; the nodes represent different HQ rMAGs and are colored according to each group of plant-specific indicators; the edge thickness is proportional to the association between nodes; (D) density plots showing the module participation of HQ rMAGs within or between each group of plant-specific indicators in the AS and AT systems over time; the vertical dashed lines indicate the mean participation of each module.

Indicator analysis was performed to identify HQ rMAGs that were strongly associated with each plant during temporal succession in the AS and AT systems, respectively. The HQ rMAGs were classified into five distinct groups of indicators based on their plant of origin (IG, TG, TV, and VNG groups) or non-specific plant origin (an “other” group comprising all non-plant-specific indicators of a specific plant). Each group of plant-specific indicators consisted of HQ rMAGs with diverse phylogenetic lineages (Fig. 3A). The plant-specific indicators accounted for a small proportion of the populations (relative abundances of 17.7%–32.6% and 36.3%–39.4% in the AS and AT systems, respectively) in each plant over time (Fig. 3B).

**Figure 4:**
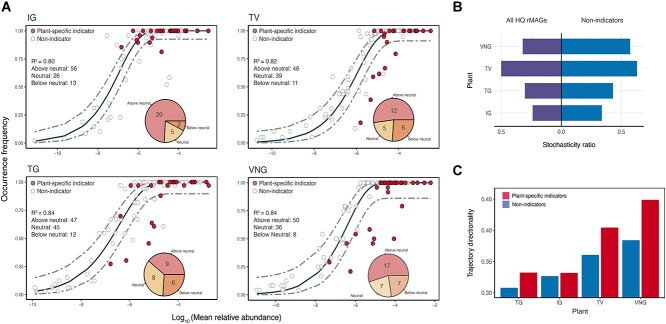
Assembly mechanism and trajectory directionality of the plant-specific indicators in the activated sludge systems; (A) fit of the Sloan neutral model used to identify the assembly mechanism of the plant-specific indicators in each plant over time; the plant-specific indicators are highlighted; the solid line shows the neutral prediction, with the 95% confidence interval denoted by the dashed lines; the goodness of fit of the model and the number of HQ rMAGs in the below, within, and above neutral expectation groups are shown; the inset pie chart shows the numbers of plant-specific indicators in the below, within, and above neutral expectation groups; (B) stochasticity of microbial communities in individual plants based on all of the HQ rMAGs or excluding the indicators; (C) trajectory directionality analysis of plant-specific indicators and non-indicators in individual plants.

A co-abundance network for all HQ rMAGs was constructed for the AS and AT systems. Intriguingly, the association network displayed a strong partitioning pattern of the microbial community members according to the groups of plant-specific indicators, indicating niche differentiation (Fig. 3C). Module participation analysis also revealed a significant and stronger association between HQ rMAGs from the same group of plant-specific indicators than between those from different groups of plant-specific indicators in both the AS and AT systems (Mann–Whitney test, both *P* < 2.2 × 10^−16^) (Fig. 3D). The Sloan neutral model was applied to query whether the plant-specific indicators were selected by environmental conditions or assembled through neutral processes in each plant over time (Fig. 4A and Fig. S10A). More than half of the plant-specific indicators—up to 81.5% and 78.7% of all indicators in the respective AS and AT systems—deviated from the neutral expectation, indicating that deterministic processes shaped the assembly of indicators. A null model-based method was used to further verify the determinism of plant-specific indicators by estimating the NSTs of the assemblies of the microbial community based on all of the HQ rMAGs or non-indicators (Fig. 4B, Figs S10B and S11). The results showed that the communities comprising all of the HQ rMAGs, which included both the plant-specific indicators and the non-indicators, exhibited a low NST (0.24–0.50 for the AS systems and 0.27–0.49 for the AT systems), indicating that deterministic processes were the dominant assembly processes that shaped the microbial community in individual plants over time. In contrast, and as expected, microbial communities excluding plant-specific indicators displayed a larger NST (0.34–0.63 for the AS systems and 0.37–0.60 for the AT systems), confirming the hypothesis that plant-specific indicators, which were selected by environmental conditions of the systems, yielded stronger determinism than other taxa. Compared with non-indicators, the plant-specific indicators exhibited a higher level of community trajectory directionality (Fig. 4C and Fig. S10C), indicating a large variation in the population of such indicators in both systems of each plant over time.

**Figure 5:**
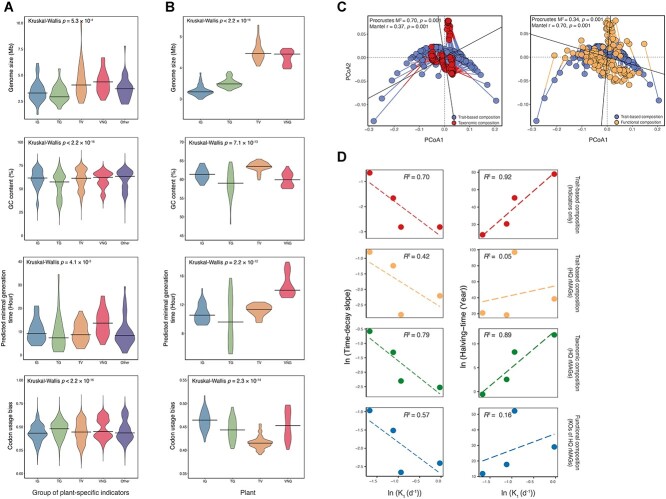
Genomic traits of microbial communities, similarities between community compositions and correlations between microbial succession, and COD removal kinetics in the activated sludge systems; (A) genomic traits of each group of plant-specific indicators; (B) CWMs of genomic traits of samples from individual plants based on plant-specific indicators; (C) Procrustes and Mantel tests of the trait-based composition with the taxonomic or functional composition; the taxonomic, functional, and trait-based composition was calculated based on the HQ rMAGs using the Bray–Curtis dissimilarity; (D) correlation between the coefficient of the first-order COD removal rate (*K*_1_) with the time-decay slope or halving-time.

An assessment of genomic traits at the species level (genome size, GC content, predicted growth rate, and codon usage bias) was conducted for each group of plant-specific indicators to elucidate the fitness and ecological niches of the HQ rMAGs. Significant differences in the four genomic traits were observed between the groups of plant-specific indicators for both the AS and AT systems (Kruskal–Wallis test, all *P* < .001) (Fig. 5A and Fig. S12A). Faster predicted growth rate, lower GC content, smaller genome size, and higher codon usage bias were observed for the HQ rMAGs of the indicators of plant TG than for those of the other plants. In contrast, the indicators of plant VNG had a slower predicted growth rate and larger genome size than the other plants. In addition, there were significant differences in the CWMs of the genomic traits of the plant-specific indicators across plants in both the AS and AT systems (Kruskal–Wallis test, all *P* < .001) (Fig. 5B and Fig. S12B); for example, plant TG had a faster CWM of predicted growth rate and lower CWM of genome size than the other plants. As in the taxonomic and functional composition results, the trait-based compositions clustered by plant in both the AS and AT systems (PERMANOVA, both *P* = .001) (Fig. S13).

The presence and absence of KOs in the HQ rMAGs were investigated to clarify the ecological niches of the HQ rMAGs in the context of their functional potential. Although the HQ rMAGs shared approximately half of the total number of KOs, their metabolic functions still clustered according to the groups of plant-specific indicators in the AS and AT systems (PERMANOVA, both *P* = .001) (Fig. S14). As expected, HQ rMAGs belonging to the different groups of plant-specific indicators shared some of the major metabolic functions, including organic carbon oxidation (100% of HQ rMAGs in both systems) and fermentation (97.9% and 98.9% of HQ rMAGs in the AS and AT systems, respectively) (Fig. S15). The denitrification function was also predicted to be broadly present in the HQ rMAGs of both systems. Overall, the distinct genomic traits and metabolic functions, coupled with the co-abundance network partitioning patterns, indicated niche differentiation between the groups of plant-specific indicators.

### Trait-based composition was stable, and community composition was correlated with treatment process kinetics during temporal succession

**Figure 6:**
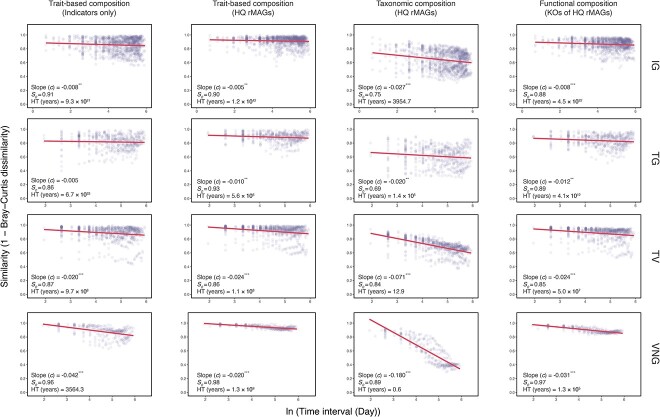
Halving-time of microbial community in the activated sludge systems; the solid line represents the decay of community similarity between the sample similarity and the time intervals; the strength of the turnover rate (slope *c*) was calculated using the logarithmic decay model (^***^P < .001, ^**^P < .01, ^*^P < .05); *S*_0_ is the initial community similarity in the shortest time interval, and halving-time (HT) is the time required for the community similarity to reduce by half.

The temporal succession of taxonomic, functional, and trait-based compositions based on all of the HQ rMAGs was determined by the time-decay slope and halving-time. Significant positive relationships were observed in the time-decay slope between all three compositional dissimilarities and time intervals for both the AS and AT systems (all *P* < .05, except for the trait-based compositions in plants TG and TV, where *P* > .05) (Figs S16 and S17). The taxonomic compositions of the AS and AT systems in individual plants had a steeper time-decay slope (0.08–0.56 for the AS systems and 0.05–0.55 for the AT systems) than both the trait-based (0.06–0.52 for the AS systems and 0.01–0.42 for the AT systems) and functional (0.07–0.38 for the AS systems and 0.03–0.51 for the AT systems) compositions, indicating extensive variations in taxa over time. The halving-times of the trait-based and functional compositions were consistently longer than those of the taxonomic compositions for both the AS and AT systems of individual plants (Fig. 6 and Fig. S18), confirming that the trait-based and functional compositions of microbial communities were stable during temporal succession.

The similarities between the trait-based composition and the taxonomic or functional compositions were examined to evaluate the degree to which trait-based variations aligned with taxonomic and functional variations over time. The trait-based compositions showed a significant and stronger correlation with functional compositions (all *P* = .001; Procrustes M^2^ = 0.34 and Mantel *r* = 0.70 for the AS systems, and Procrustes M^2^ = 0.42 and Mantel *r* = 0.84 for the AT systems) than with taxonomic compositions (all *P* = .001; Procrustes M^2^ = 0.70 and Mantel *r* = 0.37 for the AS systems, and Procrustes M^2^ = 0.71 and Mantel *r* = 0.48 for the AT systems) in both systems (Fig 5C and Fig. S12C).

The potential link between succession in community compositions and the kinetics of treatment processes was investigated to clarify the stability and succession of microbial communities in the AS systems with different treatment performances. The correlation between the first-order COD removal kinetics and the temporal succession rate in the trait-based, taxonomic, or functional compositions was estimated in the AS systems. The *R*^2^ values between the time-decay slope and *K*_1_ ranged from 0.42 to 0.79, and those between the halving-time and *K*_1_ ranged from 0.05 to 0.92 (Fig. 5D). These results suggest that the kinetics of contaminant removal or the biochemical characteristics of a treatment process can reflect the succession of microbial communities.

## Discussion

Microbial communities play a crucial role in the removal of carbon, nutrients, and other constituents in engineered systems [5, 62]. Therefore, to facilitate the derivation of effective strategies for manipulating microbial communities to improve the performance of engineered systems, it is imperative to understand fundamental microbial characteristics (e.g. genomic traits and metabolic functions) in different environmental conditions, determine the succession patterns of microbial populations, and identify the microbial community members that persist during temporal succession. As short-read-based metagenomics and low- or medium-quality MAGs lack sufficient resolution for precise annotation of taxonomy and functions and assessment of genomic traits [63, 64], we used reconstructed HQ rMAGs to investigate microbial communities. The strong plant-based partitioning in the taxonomic, functional, and trait-based compositions of HQ rMAGs in the AS and AT systems supports the hypothesis that environmental conditions of engineered systems determine the compositions of their microbial communities [65]. Compared with the taxonomic composition, the functional and trait-based compositions tended to be more stable during temporal succession, which is consistent with previous reports of higher dissimilarities in taxonomic compositions than in functional compositions of microbial communities [66, 67]. In addition, the significant and positive correlation between the taxonomic and functional compositions indicated that there was a partial functional dependency in the microbial communities, perhaps attributable to the microbes in these communities sharing some functions but differing in their ecological requirements in the treatment systems [51].

Quantification of the contributions of the replacement and richness difference processes to compositional variations showed that replacement processes largely accounted for taxonomic and functional dissimilarities, whereas richness difference processes made a negligible contribution to the variations in both the AS and AT systems. A replacement process is one in which a species or function is replaced by another due to shifts in environmental conditions or ecosystem disturbances [68, 69]. Heterogeneity in environmental conditions over time and/or between plants likely drove the observed variations in taxonomic and functional compositions across plants over time. Consistent with the β-diversity decomposition analysis, the estimated NST (< 0.5) suggests that the assembly of microbial communities in engineered systems is primarily driven by environmental conditions [70, 71].

From the perspective of microbiome management in WWTPs, it is necessary to identify and characterize specific microbial populations that are strongly associated with the environmental conditions of treatment systems and possess metabolic functions that are important for biological processes [72, 73]. Given the heterogeneity in environmental conditions across the four plants, indicator analysis was conducted to identify HQ rMAGs that were specific to the AS and AT systems of a plant. Despite the high variability in taxonomic composition between the five groups of plant-specific indicators, the majority of the functional repertoires were present across all of the groups, indicating that taxonomically distinct assemblages could perform similar metabolic functions. The partitioning in the association network of HQ rMAGs according to the groups of plant-specific indicators and the strong interactions between HQ rMAGs from the same group suggest that the members of microbial communities were differentiated into niches [74], with HQ rMAGs from the same group sharing a similar functional niche and a stronger ecological relationship than those from different groups [75]. In individual plants, the microbial communities based on all of the HQ rMAGs showed high goodness of fit (R^2^ > 0.73) to the Sloan neutral model, suggesting that dispersal contributed to community assembly over time [76]. The assessment of taxa distributions over time using the Sloan neutral model revealed that most of the plant-specific indicators deviated from the neutral expectation in individual plants, suggesting that the indicators were predominantly governed by environmental conditions over time [53, 77].

As the Sloan neutral model tends to overestimate the influence of neutral processes in community assembly, taxa falling within the neutral exception may also be driven by niche-based processes [54]. Thus, a null model method that estimates the stochasticity in community assembly was used to verify the determinism of the plant-specific indicators. This revealed that although the plant-specific indicators only accounted for a small proportion of microbial populations in each plant, removing them increased the NST in the community assembly, driving the microbial community toward a neutral assembly state. Furthermore, the trajectory directionality showed that the temporal variations in plant-specific indicators were more extensive than those in the non-indicators in each plant, indicating that environmental selection strongly determined the assembly of the indicators over time [78]. This finding is similar to those that have been reported in studies investigating spatially distributed microbial members in freshwater rivers, in which the assembly of specialist taxa was concluded to be primarily determined by niche-based processes rather than by generalist taxa [79, 80].

Given the challenges associated with fine-tuning many taxa in the microbial communities of engineered systems to improve performance [24], an alternative strategy could involve manipulating a small subset of species capable of dictating the overall profile of a community by creating optimized environmental conditions that favor them [81]. Plant-specific indicators, which substantially influence the succession and assembly of a community, are potential candidates for use in such a strategy. Prioritizing the manipulation of such indicators over other microbial community members may enable better control of the performance of engineered systems than is achievable with current approaches.

The genomic traits of microbes are commonly defined as organismal characteristics that are linked to cell fitness and functions in an environment [23]. The stronger correlations between trait-based and functional compositions, than between trait-based and taxonomic compositions, indicate that many genomic traits are widely distributed across the plants, and the changes in genomic traits reflect changes in the functions across the plants over time [82]. The weak correlation between genomic trait-based and taxonomic compositions may be attributable to adaptive evolution and horizontal gene transfer between the microbial community members [83]. Significant differences were observed in genomic traits between the groups of plant-specific indicators and in the CWMs of genomic traits between the plants. Compared with the other plants, plant TG had a higher codon usage bias and a faster predicted growth rate in its indicators, and these genomic traits had higher CWMs. This suggests that faster-growing microbes were present in plant TG than in the other plants [19, 58], which was perhaps due to the copiotrophic environment in plant TG [42], in which a mixture of industrial and municipal wastewater with a high nitrogen concentration provided rich nutrients for microbial growth.

Genomic traits may not only reflect how microbes adapt to certain environments but also determine the performance and functioning of their systems [83]. A higher first-order COD removal kinetic coefficient and higher COD removal efficiency were estimated for plant TG than for the other plants, which is consistent with the notion that fast-growing microbes typically have a high biochemical reaction rate, especially when sufficient nutrients are available, thereby enhancing the treatment performance of engineered systems [84].The genome size of the plant-specific indicators of plant TG was also smaller than that of the other plants. A small genome may facilitate rapid growth, as microbes with small genomes typically have lower nutrient requirements [85] and mutational loads [86] than those with large genomes. In contrast, the plant-specific indicators of plants IG, TV, and VNG had higher GC contents than those of plant TG; this higher GC may be a mechanism for enhancing cellular resistance to the environmental stresses imposed by higher COD loading in the three plants [87].

Overall, the microbial communities based on HQ rMAGs exhibited divergent succession in taxonomic, functional, and trait-based compositions over time in each plant. The temporal variations in taxonomic composition were greater than those in functional and genomic trait composition. This demonstrates the stability of community functions and traits over time [88] and indicates that they could serve as a set of targets for monitoring the functioning and performance of engineered systems [83].

This study has some limitations. First, our study focused primarily on characterizing the microbial populations in the WWTPs based on HQ rMAGs, and the diversity was not fully explored using the applied sequencing depth [89]. Therefore, future studies of full-scale WWTPs should employ high-depth sequencing to generate MAGs for most of the taxa, including less-abundant and rare taxa, to facilitate a more comprehensive understanding of microbial ecology in engineered systems. Second, we examined a small number of treatment plants, accompanied by a limited set of measured environmental parameters, and some key parameters, such as solids retention time and concentration of volatile suspended solids, were missing. Thus, future investigations must examine samples from a greater number of diverse types of engineered systems in conjunction with measurements of a broad set of environmental parameters to facilitate a more comprehensive understanding of the relationships between the genomic and functional traits of taxa and the kinetics of specific biological processes, such as nitrification, denitrification, and phosphorus removal. Additionally, an analysis based on a broader sample would verify the stability of genomic traits over time in engineered systems. Third, although we examined genomic traits at the species and community levels, only four genomic traits were considered. Thus, additional genomic traits, such as the number of rRNA gene copies and regulatory genes, should be considered in future studies. Lastly, a multi-omics study that includes metagenomics together with metatranscriptomics and metaproteomics is required to characterize the genomic traits and ecological niches of active microbes and their expressed metabolic functions in engineered systems.

In this study, we discovered niche differentiation and genomic trait variations between microbial community members and found that plant-specific indicators contributed to niche-based assembly of microbial communities. Moreover, we found that functional and trait-based compositions were stable over time and that the succession of taxonomic, functional, and trait-based compositions was associated with substrate removal kinetics. The abovementioned findings provide insights into the ecological niches of microbial community members and the ecological mechanisms governing community variations over time in engineered systems.

## Supplementary Material

SI_text_figure_paged_wrae042

SI_tables_wrae042

## Data Availability

The raw sequences were deposited into the National Center for Biotechnology Information Sequence Read Archive under BioProject accession number PRJNA996624. The contigs of each HQ rMAG were deposited in the public FigShare repository (http://dx.doi.org/10.6084/m9.figshare.24623736). The other data that support the findings of this study are available from the corresponding author upon reasonable request.
