# Supplementary material for: Niche differentiation in microbial communities with stable genomic traits over time in engineered systems
Source: ISME J. 2024 Mar 12;18(1):wrae042. doi: 10.1093/ismejo/wrae042 (PMC10987969; doi:10.1093/ismejo/wrae042)
Supplement: SI_text_figure_paged_wrae042 [file si_text_figure_paged_wrae042.pdf]

Supplementary Information for

Niche differentiation in microbial communities with stable genomic traits over time in engineered systems

Jinjin Yu,[1] Justin Y. Y. Lee,[1] Siang Nee Tang,[2] and Patrick K. H. Lee[3]*

[1]School of Energy and Environment, City University of Hong Kong, Hong Kong SAR, China

[2]Facility Management and Environmental Engineering, TAL Group, Hong Kong SAR, China

[3]School of Energy and Environment and State Key Laboratory of Marine Pollution, City University of Hong Kong, Hong Kong SAR, China

Correspondence: *B5423, Yeung Kin Man Academic Building, School of Energy and Environment, City University of Hong Kong, Tat Chee Avenue, Kowloon, Hong Kong SAR, China; E-mail: patrick.kh.lee@cityu.edu.hk; Tel: (852) 3442-4625; Fax: (852) 3442-0688.

Supplemental information includes:

Supplementary Text S1

References

Supplementary Table S1 to S4

Supplementary Figure S1 to S18

Text S1

Detailed materials and methods

Kinetics modeling

The first-order [1] and Grau second-order [2] models were applied to the combined temporal COD data of all of the AS systems, and the model giving the best fit was adopted to estimate the COD removal kinetics of the AS system of each plant. The first-order model is $-\frac{dS}{dt} = \frac{QS_i}{V} - \frac{QS_e}{V} - K_1 S_e$, where S_i and S_e are the influent and effluent COD concentration, respectively; Q is the influent flow rate, V is the volume of the tank, and K_1 is the coefficient of the first-order COD removal rate. Under pseudo-steady-state conditions with the rate of change of COD concentration (dS/dt) being negligible, the model simplifies to $K_1 S_e = \frac{Q(S_i - S_e)}{V}$. The Grau second-order model is $-\frac{dS}{dt} = \left(\frac{S_e}{S_i}\right)^2 K_2 X_b$, where K_2 is the coefficient of the second-order COD removal rate and X_b is the biomass concentration. After linearization, the model simplifies to $\frac{VS_i}{Q(S_i - S_e)} = \frac{V}{Q} + \frac{S_i}{K_2 X}$.

The fits of the two kinetics models were evaluated by linear modeling using the R package lme4 (v1.1-32), with plant as the additional fixed effect. Specifically, for the first-order model, the linear model structure was $Q(S_i - S_e)/V \sim S_e$ + plant; for the Grau second-order model, the linear model structure was $VS_i/Q(S_i\text{-}S_e) \sim V/Q$ + plant [3, 4]. The model with the higher R^2 value was utilized to estimate the COD removal kinetics in the AS system of each plant using the R package nls.mutltstart (v1.2.0).

Sample collection and metagenomic sequencing

From October 2018 to October 2019, samples were collected from the AS and AT tanks of the four plants (**Figure S1a**). Planktonic wastewater samples (500 mL) were collected biweekly from the well-mixed tanks by filtration (0.22 µm, 47 mm; Durapore, Germany), and

biofilm samples were obtained monthly by scraping ~50 g of material from the cotton carriers. Additionally, two months of intensive sampling (daily to weekly) were carried out at plant IG to obtain planktonic samples (from December 2018 to January 2019). All of the samples were stored at −80°C until genomic DNA was extracted using the DNeasy PowerSoil Kit (Qiagen, Germantown, MD, USA) according to the manufacturer's instructions. Three new autoclaved filters were processed in parallel with the samples and served as negative controls.

Metagenomic sequencing of a total of 146 AS and 186 AT samples from the four WWTPs was performed according to the manufacturer's workflow on an Illumina NovaSeq platform to generate 150-bp paired-end reads (Novogene, China). An average of ~14.8 million raw paired-end reads were generated per AS or AT sample. The quality of the sequencing reads was evaluated using FastQC [5] (v0.11.5), and illumina-utils [6] (v2.4.1) with the default parameters was used for quality filtering. An average of ~14.3 million paired-end reads per sample remained after quality control. In contrast, the three negative controls yielded only ~58,000 paired-end reads per sample, which was two-to-three orders of magnitude lower than the WWTP samples. Due to the negligible number of reads generated in the negative controls, no further decontamination procedures were deemed necessary for the AS and AT samples.

Microbial community assembly mechanism and trajectory directionality

The Sloan neutral model [7], which predicts the relationship between the occurrence frequencies of taxa and their relative abundances across meta-communities, was used to determine the assembly mechanisms of plant-specific indictors and non-indicators in individual plants during temporal succession. Based on the deviation from the 95% confidence interval around the neutral prediction, all of the HQ rMAGs of each plant were classified into the three predicted partitions: above neutral, below neutral, or neutral. The normalized stochasticity ratio (NST) of the microbial communities, based on all of the HQ rMAGs or non-indicators in each

plant, were quantified using a null model-based approach in the R package NST [8] (v3.0.6). A NST greater than 0.5 indicates that a community is likely to be neutral. Trajectory directionality analysis of the plant-specific indicators and non-indicators in individual plants was performed based on the Bray–Curtis dissimilarity in the R package ecotraj (v0.1.0) to evaluate the temporal dynamics of microbial community members. In the trajectory directionality analysis, random sampling was applied to the non-indicators to ensure that the sample size between the plant-specific indicators and non-indicators was the same.

Community weighted means (CWMs) of genomic traits and trait-based composition

Genomic traits at the community level of samples from each plant were calculated using all of the plant-specific indicators and the CWMs method [9] as follows: $\sum_{i=1}^{S} p_i\, x_i$, where p_i is the relative abundance of the plant-specific indicator *i* ($i = 1, 2, \ldots, S$), and x_i is the trait value of plant-specific indicator *i*. Furthermore, the dissimilarity in trait-based compositions between samples was calculated based on the CWMs of the four genomic traits of plant-specific indicators or all HQ rMAGs using the Bray–Curtis dissimilarity. Specifically, the trait-based composition dissimilarity was estimated by first normalizing the values of genomic traits, then summing the CWMs of all four genomic traits, and finally determining the Bray–Curtis dissimilarity distance between two communities [9].

Time-decay slope and halving-time

The temporal succession of taxonomic, functional, and trait-based compositions in each plant were evaluated by the time-decay slope [10] and halving-time [11]. The time-decay slope is an estimated rate of succession per unit time that is obtained via a linear regression equation that models the relationship between logarithm community dissimilarity (*DS*) and logarithm time interval (*T*) according to $\log DS = a + b \log_{10}(T)$, where *T* is the difference between two

sampling dates, *a* is the intercept, and *b* represents the succession rate. A high value of *b* indicates a rapid succession.

The halving-time is an estimate of the time at which the community similarity becomes half of what it was initially. This metric was determined using the logarithmic decay model $S = c \ln(T) + int$, where *S* represents the community similarity in a time interval (i.e., the difference between two sampling dates; denoted as *T*), *c* is the rate of time decay, and *int* is the intercept of the model. By assuming a community similarity of 1 when the time interval is 0, the corresponding halving-time (*HT*) is $HT = e^{\left(\frac{\frac{S_0}{2} - int}{c}\right)}$, where S_0 represents the initial community similarity in the shortest time interval [12]. A long halving time indicates a slow succession over time.

References

1. Borghei S, Sharbatmaleki M, Pourrezaie P, Borghei G. Kinetics of organic removal in fixed-bed aerobic biological reactor. Bioresour Technol 2008; **99**: 1118-1124.

2. Grau P, Dohanyos M, Chudoba J. Kinetics of multicomponent substrate removal by activated sludge. Water Res 1975; **9**: 637-642.

3. Shahzad H, Khan S, Habib Z. Performance evaluation and substrate removal kinetics in a thermophilic anaerobic moving bed biofilm reactor for starch degradation. Water Pract Technol 2022; **17**: 157-166.

4. Tang S, Xu Z, Liu Y, Yang G, Mu J, Jin R et al. Performance, kinetics characteristics and enhancement mechanisms in anammox process under Fe (II) enhanced conditions. Biodegradation 2020; **31**: 223-234.

5. Andrews S. FastQC: a quality control tool for high throughput sequence data. 2010. http://www.bioinformatics.babraham.ac.uk/projects/fastqc.

6. Eren AM, Vineis JH, Morrison HG, Sogin ML. A filtering method to generate high quality short reads using Illumina paired-end technology. PloS One 2013; **8**: e66643.

7. Sloan WT, Lunn M, Woodcock S, Head IM, Nee S, Curtis TP. Quantifying the roles of immigration and chance in shaping prokaryote community structure. Environ Microbiol 2006; **8**: 732-740.

8. Ning D, Deng Y, Tiedje JM, Zhou J. A general framework for quantitatively assessing ecological stochasticity. Proc Natl Acad Sci USA 2019; **116**: 16892-16898.

9. Guittar J, Shade A, Litchman E. Trait-based community assembly and succession of the infant gut microbiome. Nat Commun 2019; **10**: 512.

10. Nekola JC, White PS. The distance decay of similarity in biogeography and ecology. J Biogeogr 1999; **26**: 867-878.

11. Soininen J, McDonald R, Hillebrand H. The distance decay of similarity in ecological communities. Ecography 2007; **30**: 3-12.

12. Villarino E, Watson JR, Jonsson B, Gasol JM, Salazar G, Acinas SG et al. Large-scale ocean connectivity and planktonic body size. Nat Commun 2018; **9**: 142.

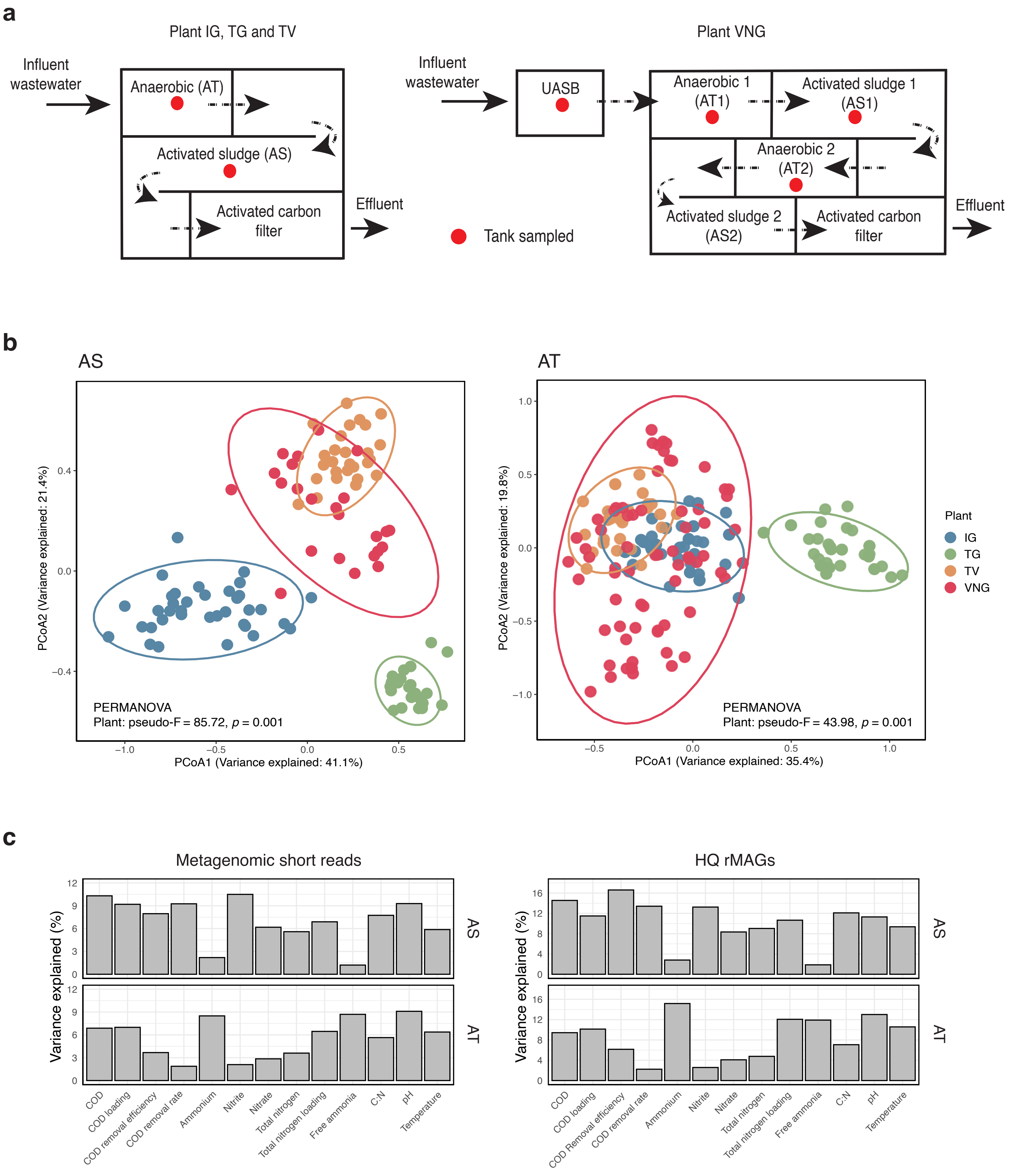

Fig. S1 Schematic diagram of the wastewater treatment plants and environemental condtiondions of the activated sludge (AS) and anaerobic treatment (AT) systems in four plants. (a) Schematic diagram of the wastewater treatment system setup. **(b)** Principal coordinate analysis (PCoA) of environmental conditions of the samples from the respective AS and AT systems in the four plants over time. In the PCoA, the points are colored according to plants and ellipses are colored based on the multivariate normal distribution at a 95% confidence interval for each plant. **(c)** Assessment of the effects of individual environmental parameters on the community variations of AS and AT systems using metagenomic short reads and high-quality representative metagenome-assembled genomes (HQ rMAGs) through PERMANOVA analysis.

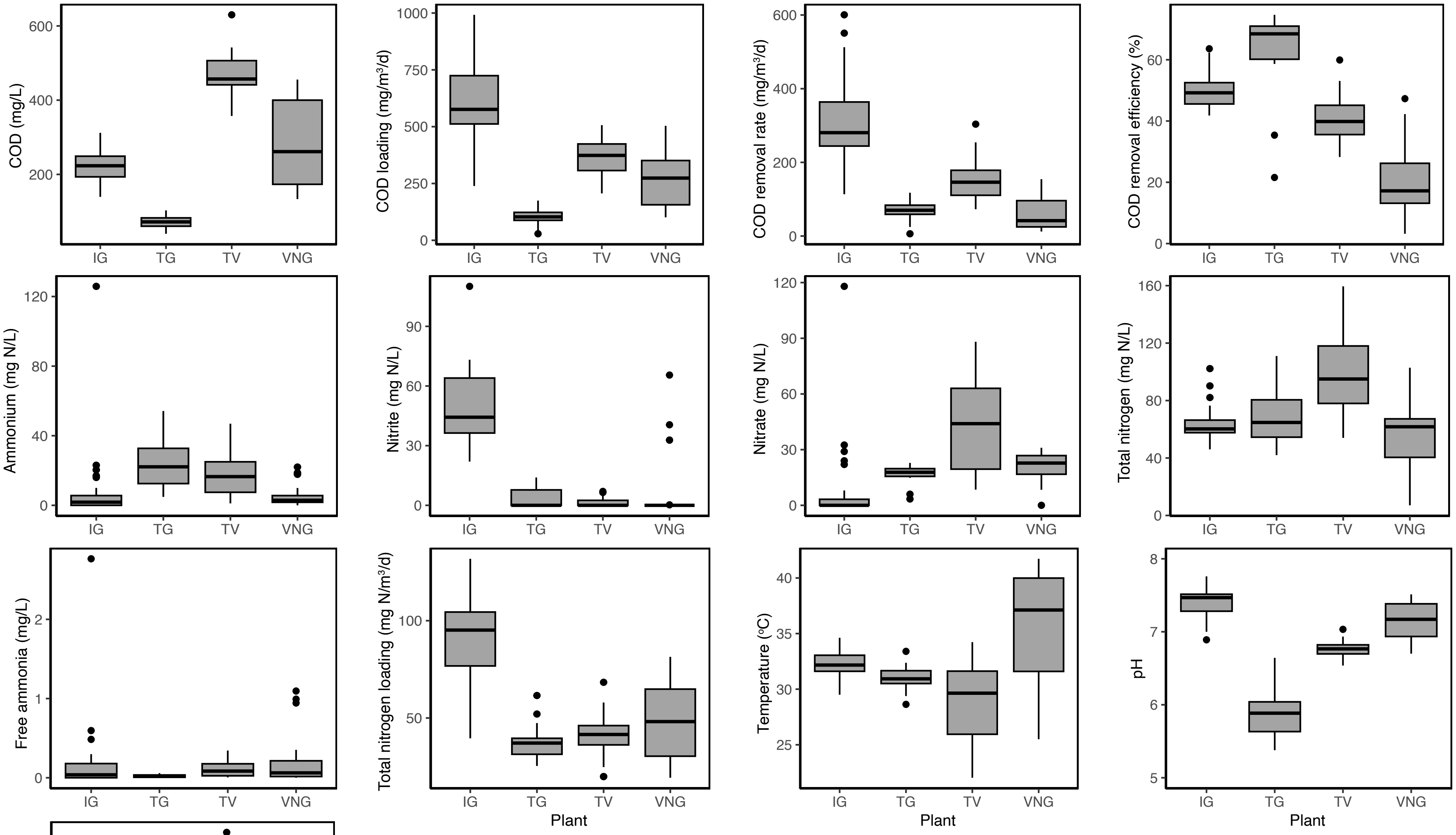

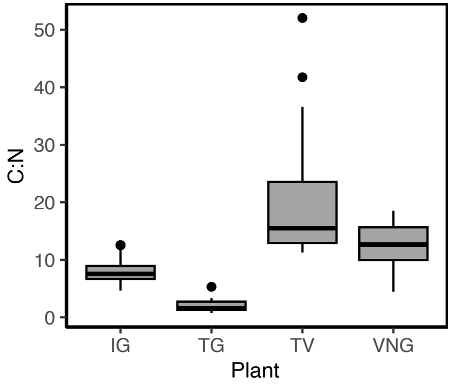

Fig. S2 Summary of the 13 environmental parameters of individual plants over time in the activated sludge systems. Boxplots represent the median, first quartile, and third quartile, and whiskers indicate the 1.5 interquartile range. Points outside the whiskers represent outliers.

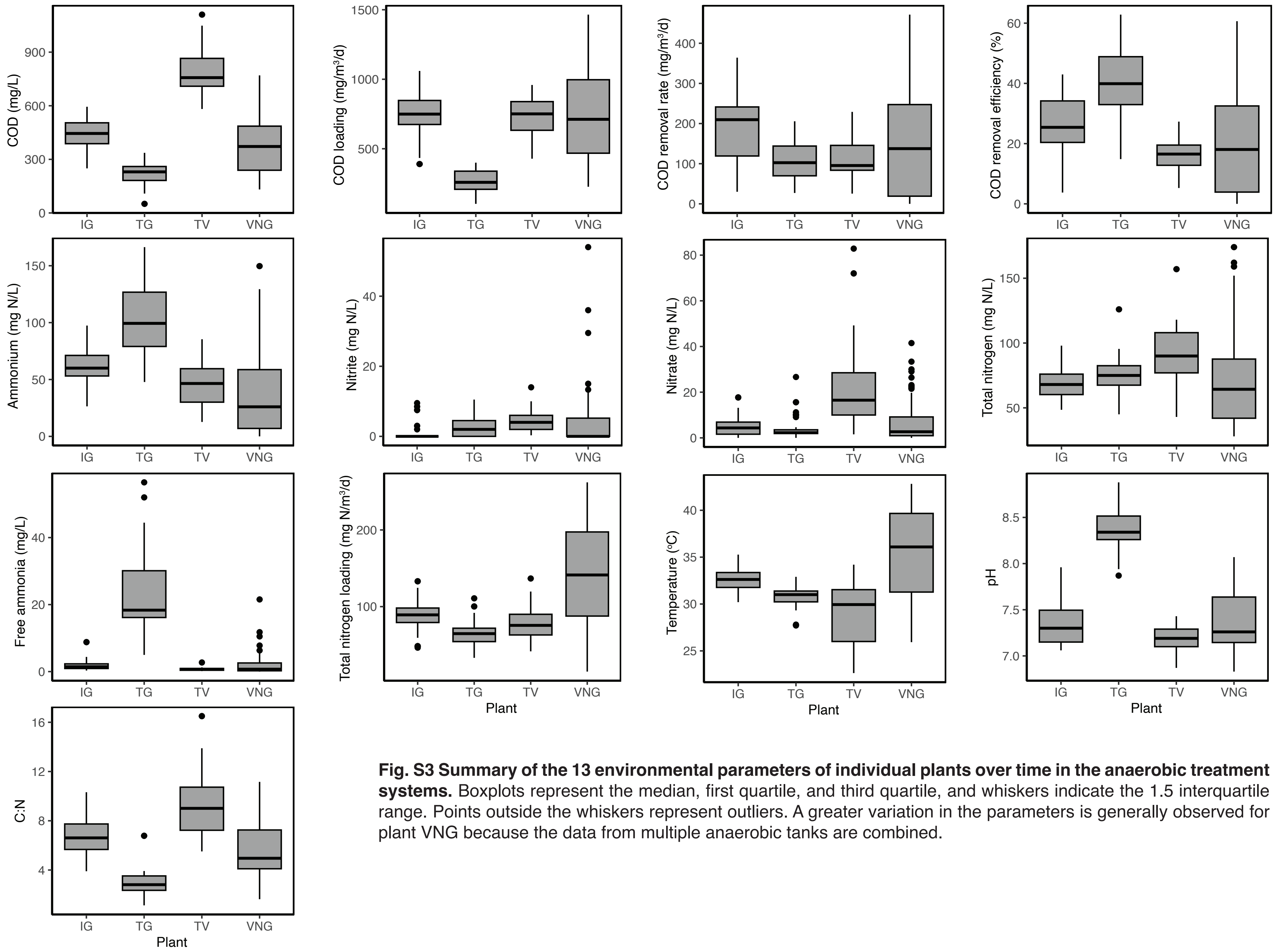

Fig. S3 Summary of the 13 environmental parameters of individual plants over time in the anaerobic treatment systems. Boxplots represent the median, first quartile, and third quartile, and whiskers indicate the 1.5 interquartile range. Points outside the whiskers represent outliers. A greater variation in the parameters is generally observed for plant VNG because the data from multiple anaerobic tanks are combined.

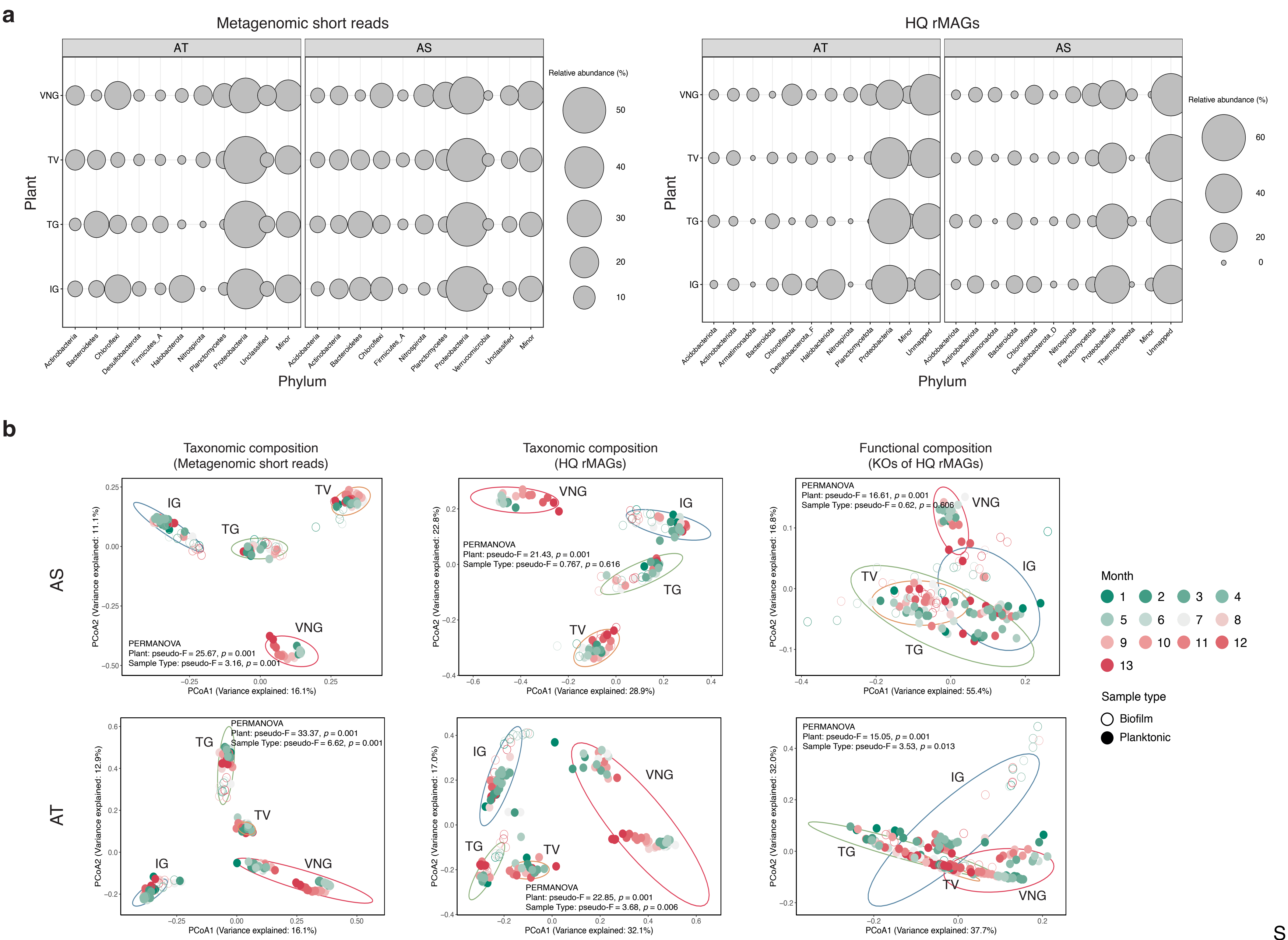
a
Metagenomic short reads
HQ rMAGs
AT
AS
Plant
VNG
TV
TG
IG
Relative abundance (%)
Phylum
Actinobacteria
Bacteroidetes
Chloroflexi
Desulfobacterota
Firmicutes_A
Halobacterota
Nitrospirota
Planctomycetes
Proteobacteria
Unclassified
Minor
Acidobacteria
Verrucomicrobia
Acidobacteriota
Actinobacteriota
Armatimonadota
Bacteroidota
Chloroflexota
Desulfobacterota_F
Desulfobacterota_D
Planctomycetota
Thermoproteota
Unmapped
b
Taxonomic composition (Metagenomic short reads)
Taxonomic composition (HQ rMAGs)
Functional composition (KOs of HQ rMAGs)
PERMANOVA
Plant: pseudo-F = 25.67, p = 0.001
Sample Type: pseudo-F = 3.16, p = 0.001
PCoA1 (Variance explained: 16.1%)
PCoA2 (Variance explained: 11.1%)
PERMANOVA
Plant: pseudo-F = 21.43, p = 0.001
Sample Type: pseudo-F = 0.767, p = 0.616
PCoA1 (Variance explained: 28.9%)
PCoA2 (Variance explained: 22.8%)
PERMANOVA
Plant: pseudo-F = 16.61, p = 0.001
Sample Type: pseudo-F = 0.62, p = 0.606
PCoA1 (Variance explained: 55.4%)
PCoA2 (Variance explained: 16.8%)
PERMANOVA
Plant: pseudo-F = 33.37, p = 0.001
Sample Type: pseudo-F = 6.62, p = 0.001
PCoA1 (Variance explained: 16.1%)
PCoA2 (Variance explained: 12.9%)
PERMANOVA
Plant: pseudo-F = 22.85, p = 0.001
Sample Type: pseudo-F = 3.68, p = 0.006
PCoA1 (Variance explained: 32.1%)
PCoA2 (Variance explained: 17.0%)
PERMANOVA
Plant: pseudo-F = 15.05, p = 0.001
Sample Type: pseudo-F = 3.53, p = 0.013
PCoA1 (Variance explained: 37.7%)
PCoA2 (Variance explained: 32.0%)
Month
Sample type
Biofilm
Planktonic

Fig. S4 The top-10 most abundant classified taxa and the temporal taxonomic and functional compositions of the activated sludge (AS) and anaerobic treatment (AT) systems in the four plants. (a) Relative abundances of the top-10 phyla based on metagenomic short reads and of the top-10 phyla based on high-quality representative metagenome-assembled genomes (HQ rMAGs) in the AS and AT systems over time. The other taxa are grouped under "Minor". **(b)** Principal coordinate analysis of the temporal taxonomic compositions (based on metagenomic short reads and HQ rMAGs) and functional compositions (based on KOs of HQ rMAGs). Points are colored by the month of sampling, and filled symbols and open symbols indicate planktonic and biofilm samples, respectively. Ellipses are colored based on the multivariate normal distribution at a 95% confidence interval for each plant.

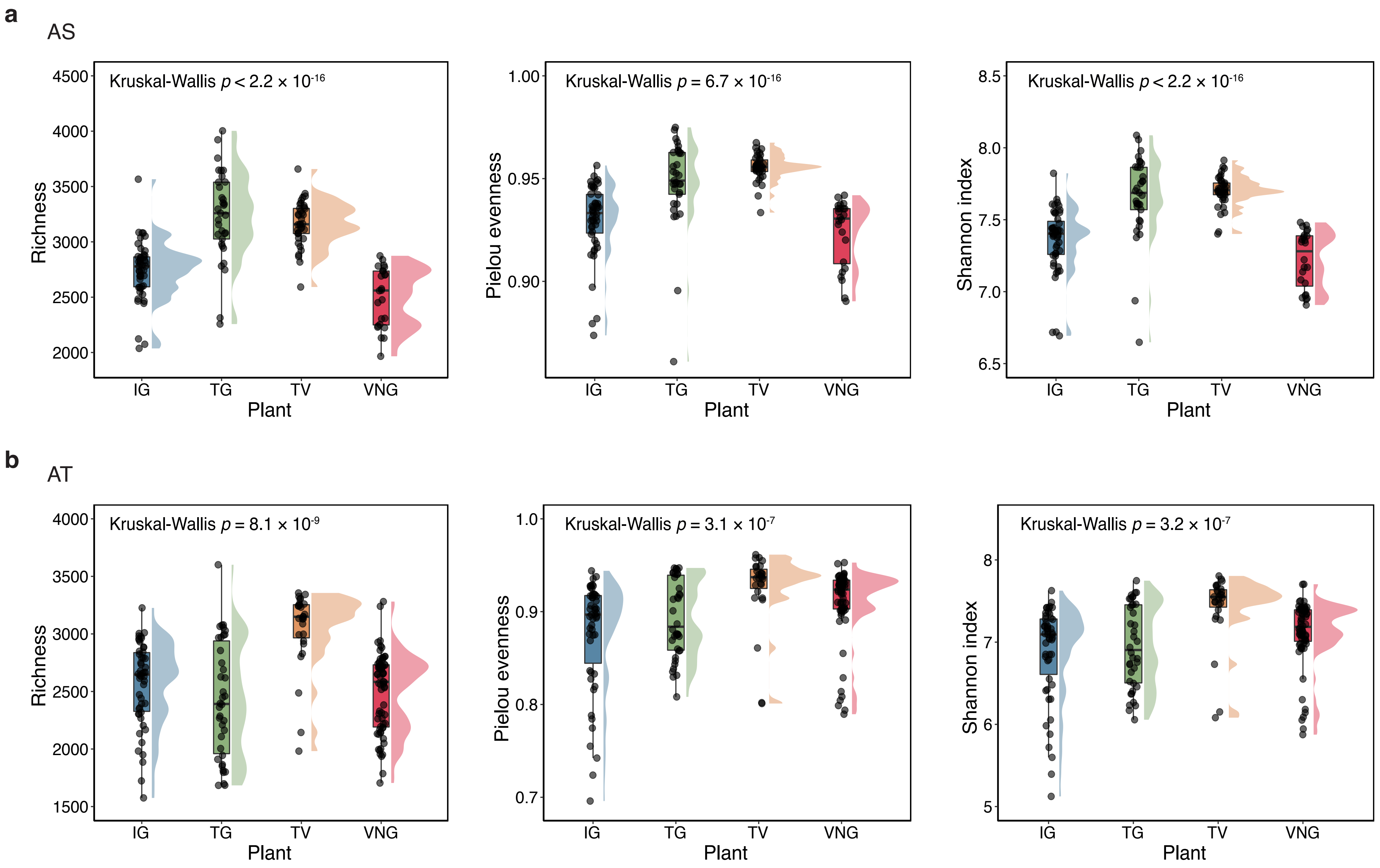

Fig. S5 α-diversity of the microbial communities in the (a) activated sludge and (b) anaerobic treatment systems of the four plants. Each point represents a sample.

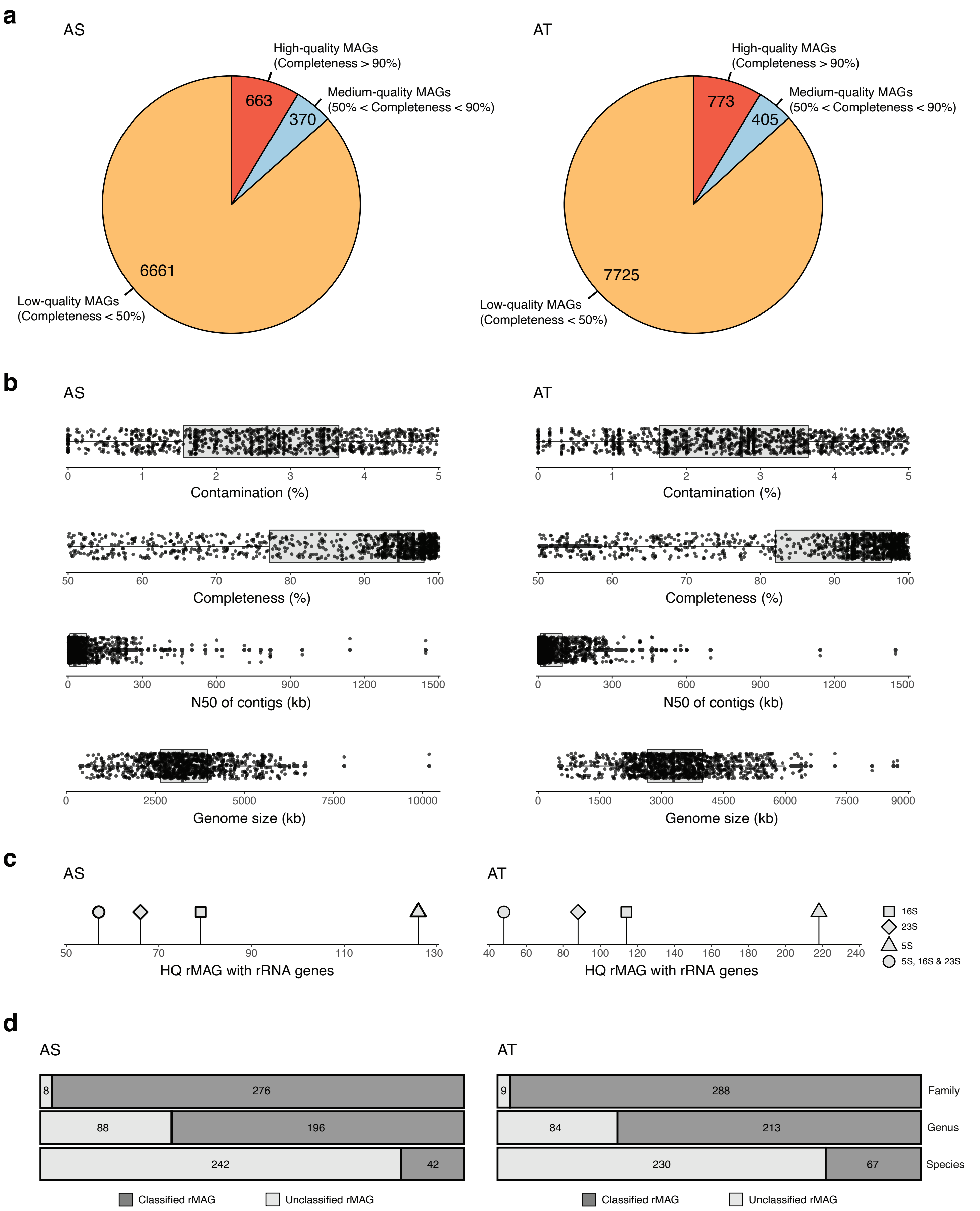

Fig. S6 Quality of the metagenome-assembled genomes (MAGs) and the numbers of classified and unclassified representative metagenome-assembled genomes (rMAGs) recovered from the activated sludge and anaerobic treatment systems. (a) Numbers of low-, medium-, and high-quality MAGs recovered. **(b)** Distribution of the quality metrics of the medium- and high-quality MAGs recovered. Each point represents a MAG. **(c)** Number of high-quality rMAGs whose 5S, 16S, and/or 23S rRNA genes were detected. **(d)** Number of classified and unclassified medium- and high-quality rMAGs based on GTDB-Tk at various taxonomic ranks.

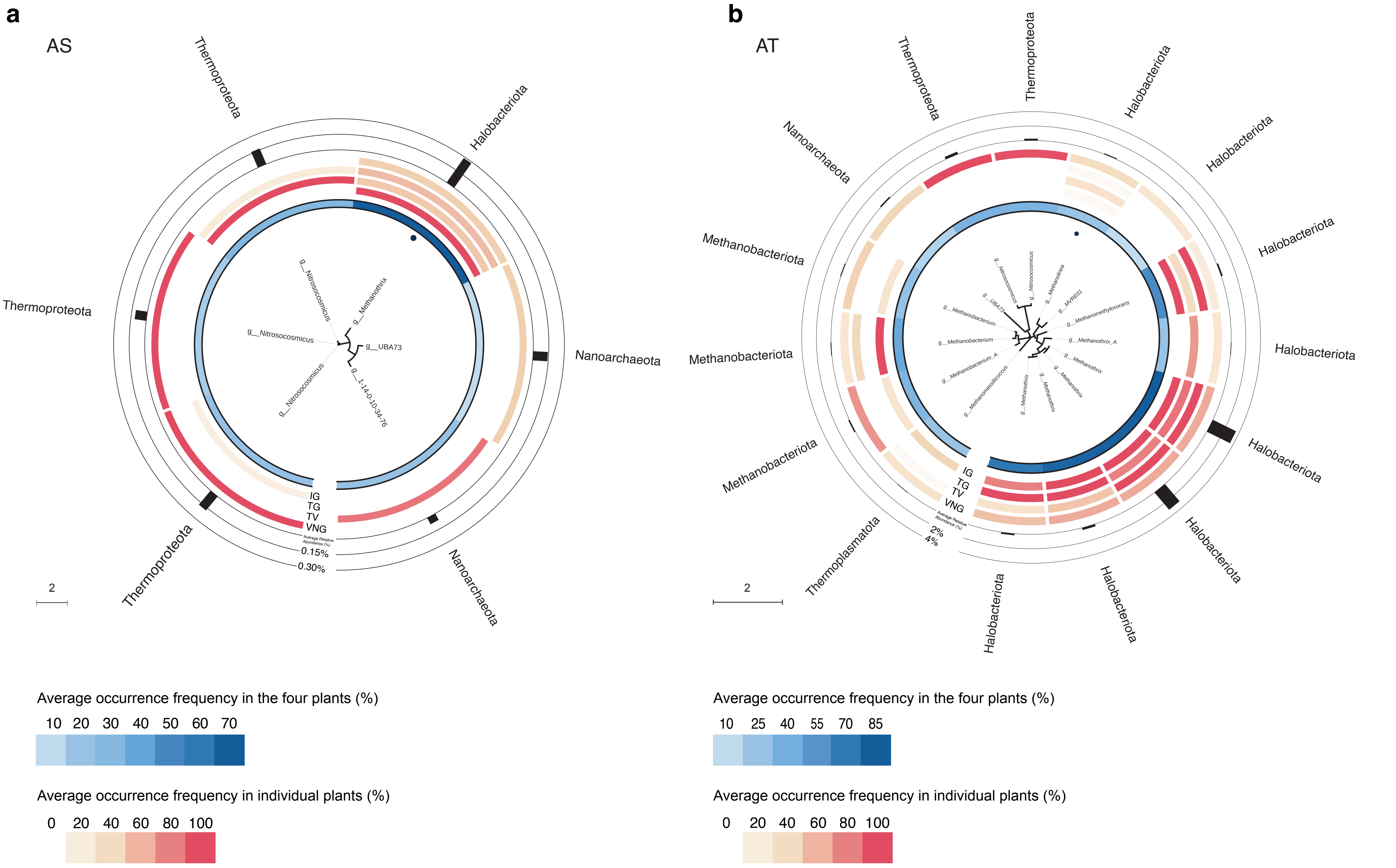

Fig. S7 Phylogenetic tree of the medium- and high-quality representative metagenome-assembled genomes (rMAGs) in archaeal lineages from the (a) activated sludge (AS) and (b) anaerobic treatment (AT) systems. The innermost ring shows the lowest possible taxonomic rank of the rMAGs (six from AS and 15 from AT) based on GTDB-Tk at the genus (prefix "g") level, and the outermost ring shows the phylum rank. High-quality rMAGs containing all of the 5S, 16S, and 23S rRNA genes and more than 18 tRNAs are highlighted with a black circle in the innermost ring. Scale bar indicates the tree scale. The average occurrence frequencies (in individual plants and the four plants) and average relative abundances in the four plants for the rMAGs over time are indicated by the heatmap and bar chart in the outer rings, respectively.

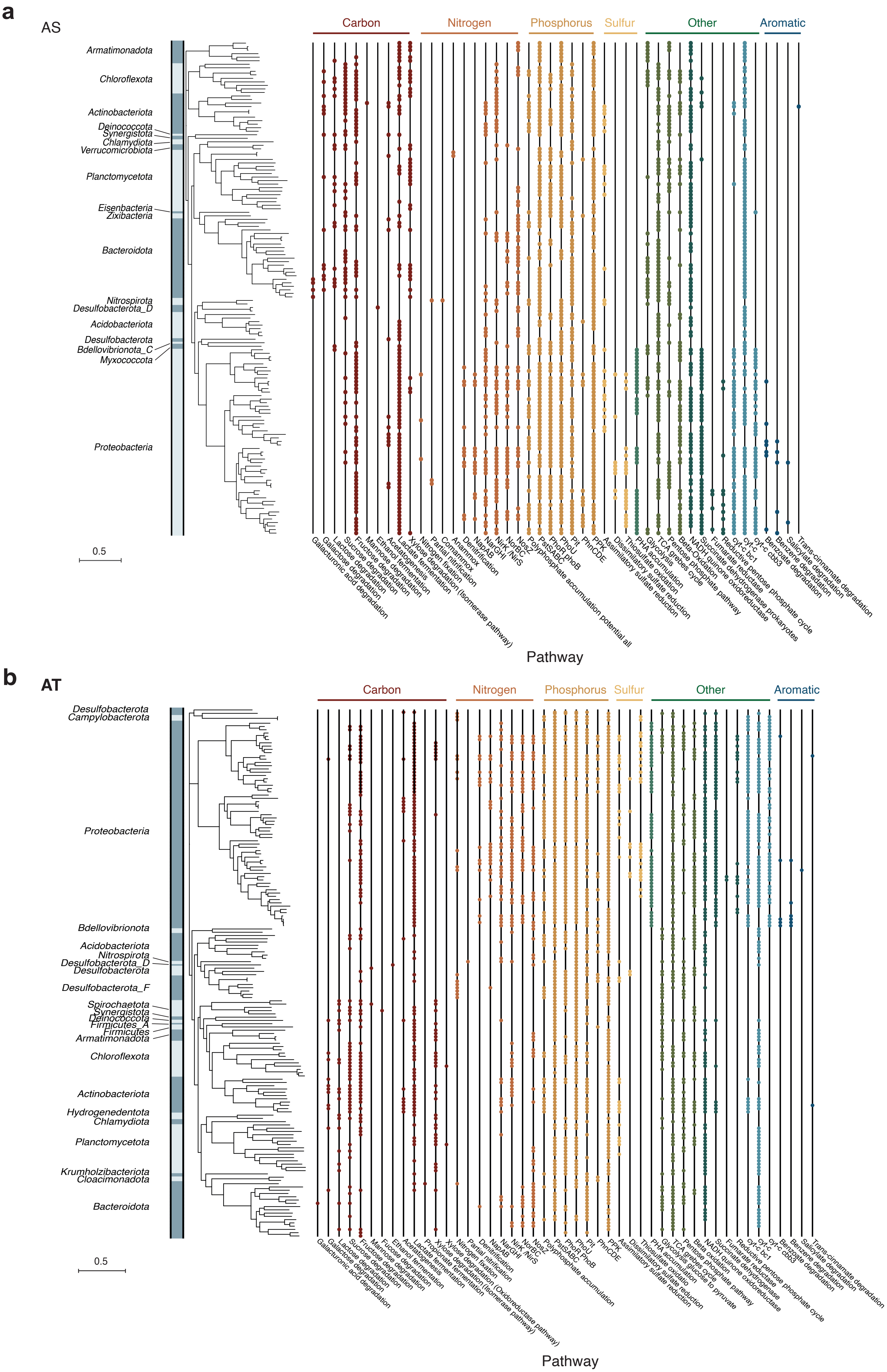

a
AS
Carbon
Nitrogen
Phosphorus
Sulfur
Other
Aromatic
Armatimonadota
Chloroflexota
Actinobacteriota
Deinococcota
Synergistota
Chlamydiota
Verrucomicrobiota
Planctomycetota
Eisenbacteria
Zixibacteria
Bacteroidota
Nitrospirota
Desulfobacterota_D
Acidobacteriota
Desulfobacterota
Bdellovibrionota_C
Myxococcota
Proteobacteria
0.5
Galacturonic acid degradation
Galactose degradation
Lactose degradation
Sucrose degradation
Fructose degradation
Mannose degradation
Ethanol fermentation
Acetogenesis
Lactate fermentation
Xylose degradation (Isomerase pathway)
Nitrogen fixation
Partial nitrification
Comammox
Anammox
Denitrification
NapAB
NarGHI
NirK/NirS
NorBC
NosZ
Polyphosphate accumulation potential all
PstSABC
PhoR PhoB
PhoU
Pit
PhnCDE
PPK
Assimilatory sulfate reduction
Dissimilatory sulfate reduction
Thiosulfate oxidation
PHA accumulation
Glycolysis
TCA krebs cycle
Pentose phosphate pathway
Beta-Oxidation
NADH quinone oxidoreductase
Succinate dehydrogenase prokaryotes
Fumarate reductase
Reductive pentose phosphate cycle
cyt-c bc1
cyt-c
cyt-c cbb3
Benzoate degradation
Benzene degradation
Salicylate degradation
Trans-cinnamate degradation
Pathway
b
AT
Carbon
Nitrogen
Phosphorus
Sulfur
Other
Aromatic
Desulfobacterota
Campylobacterota
Proteobacteria
Bdellovibrionota
Acidobacteriota
Nitrospirota
Desulfobacterota_D
Desulfobacterota
Desulfobacterota_F
Spirochaetota
Synergistota
Deinococcota
Firmicutes_A
Firmicutes
Armatimonadota
Chloroflexota
Actinobacteriota
Hydrogenedentota
Chlamydiota
Planctomycetota
Krumholzibacteriota
Cloacimonadota
Bacteroidota
0.5
Galacturonic acid degradation
Galactose degradation
Lactose degradation
Sucrose degradation
Fructose degradation
Mannose degradation
Fucose degradation
Ethanol fermentation
Acetogenesis
Lactate fermentation
Propionate fermentation
Xylose degradation (Isomerase pathway)
Xylose degradation (Oxidoreductase pathway)
Nitrogen fixation
Partial nitrification
Denitrification
NapAB
NarGHI
NirK/NirS
NorBC
NosZ
Polyphosphate accumulation
PstSABC
PhoR PhoB
PhoU
Pit
PhnCDE
PPK
Assimilatory sulfate reduction
Dissimilatory sulfate reduction
Thiosulfate oxidation
PHA accumulation
Glycolysis glucose to pyruvate
TCA krebs cycle
Pentose phosphate pathway
Beta oxidation
NADH quinone oxidoreductase
Succinate dehydrogenase
Fumarate reductase
Reductive pentose phosphate cycle
cyt-c bc1
cyt-c
cyt-c cbb3
Benzoate degradation
Benzene degradation
Salicylate degradation
Trans-cinnamate degradation
Pathway

Fig. S8 Profiles of the potential functions of the high-quality representative metagenome-assembled genomes (HQ rMAGs) in bacterial lineages from the (a) activated sludge and (b) anaerobic treatment systems. Complete pathways are indicated by solid circles, and the categories of pathways are color-coded. The bar chart on the left indicates the taxonomy of HQ rMAGs at the phylum level. Scale bar indicates the tree scale.

a

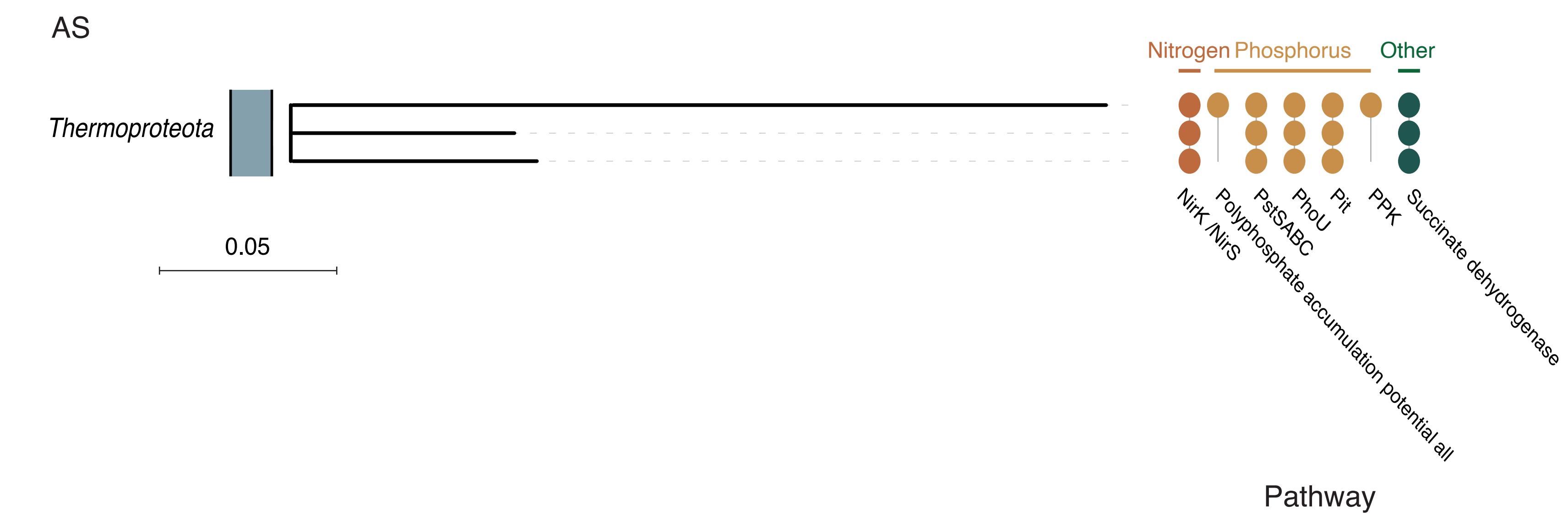

b

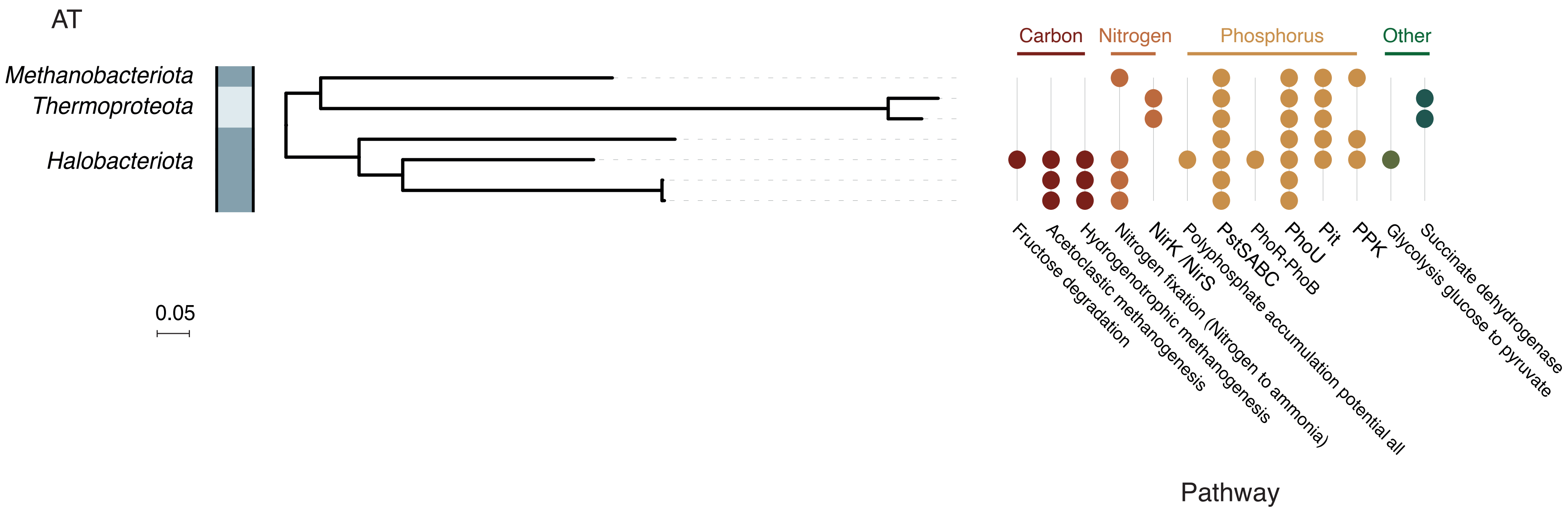

Fig. S9 Profiles of the potential functions of the high-quality representative metagenome-assembled genomes (HQ rMAGs) in archaeal lineages from the (a) activated sludge and (b) anaerobic treatment systems. Complete pathways are indicated by solid circles, and the categories of pathways are color-coded. The bar chart on the left indicates the taxonomy of HQ rMAGs at the phylum level. Scale bar indicates the tree scale.

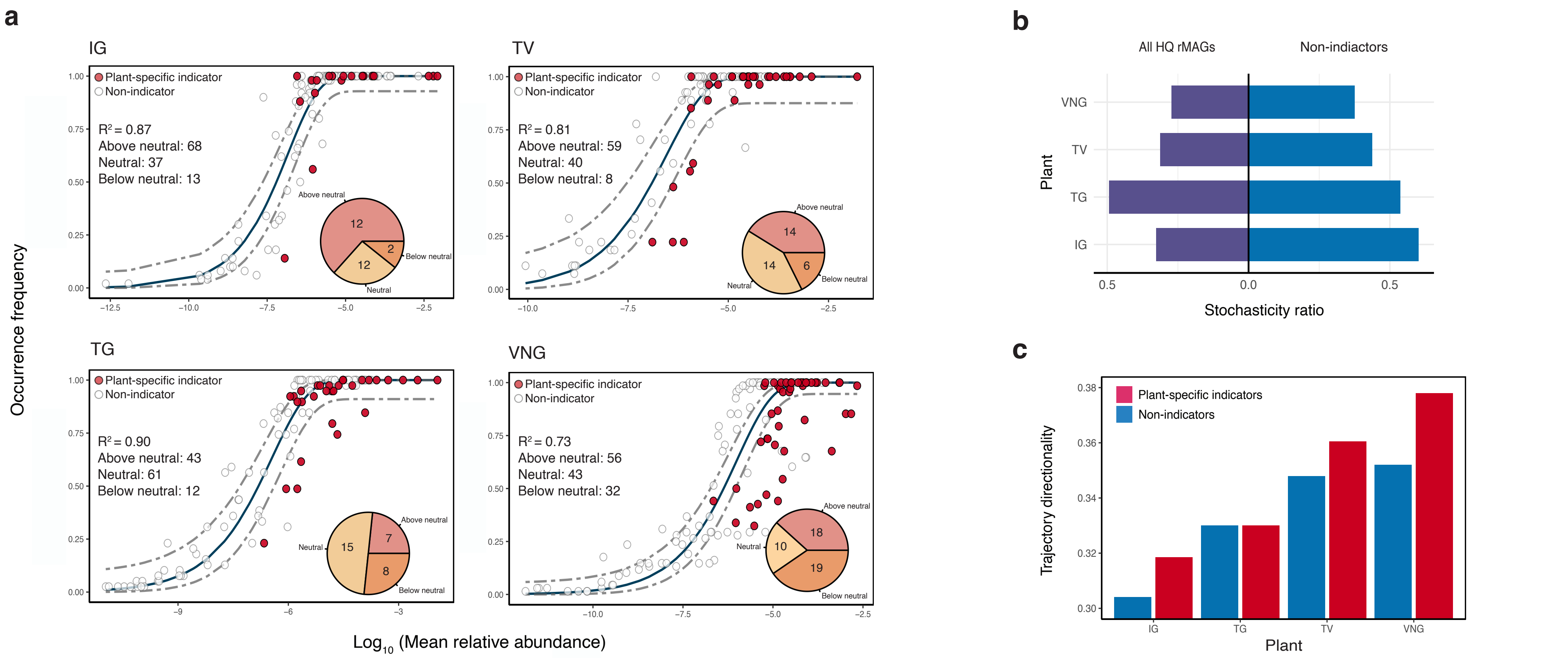

Fig. S10 Assembly mechanism and trajectory directionality of plant-specific indicators in the anaerobic treatment systems. (a) Fit of the Sloan neutral model to identify the assembly mechanism of the plant-specific indicators in the respective plants over time. The plant-specific indicators are colored in red. The solid line shows the neutral prediction, with the 95% confidence interval denoted by the dashed lines. The goodness of fit of the model and the number of HQ rMAGs in the below, within, and above neutral expectation groups are shown. The inset pie chart shows the numbers of plant-specific indicators in the below, within, and above neutral expectation groups. **(b)** Stochasticity of microbial communities in individual plants based on all of the high-quality representative metagenome-assembled genomes (purple bars) or excluding the indicators (blue bars). **(c)** Trajectory directionality analysis of plant-specific indicators (red bars) and non-indicators (blue bars) in individual plants.

a

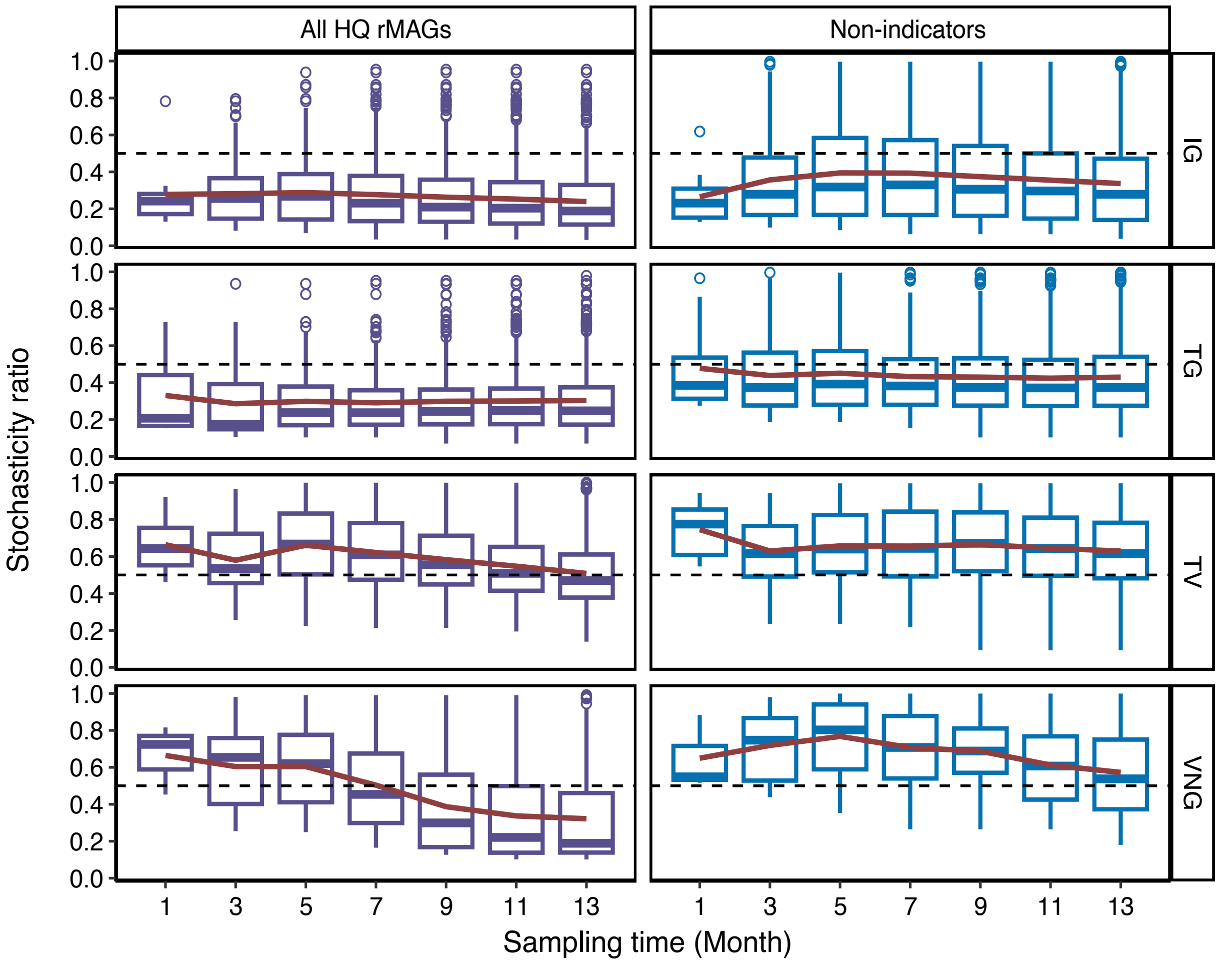

b

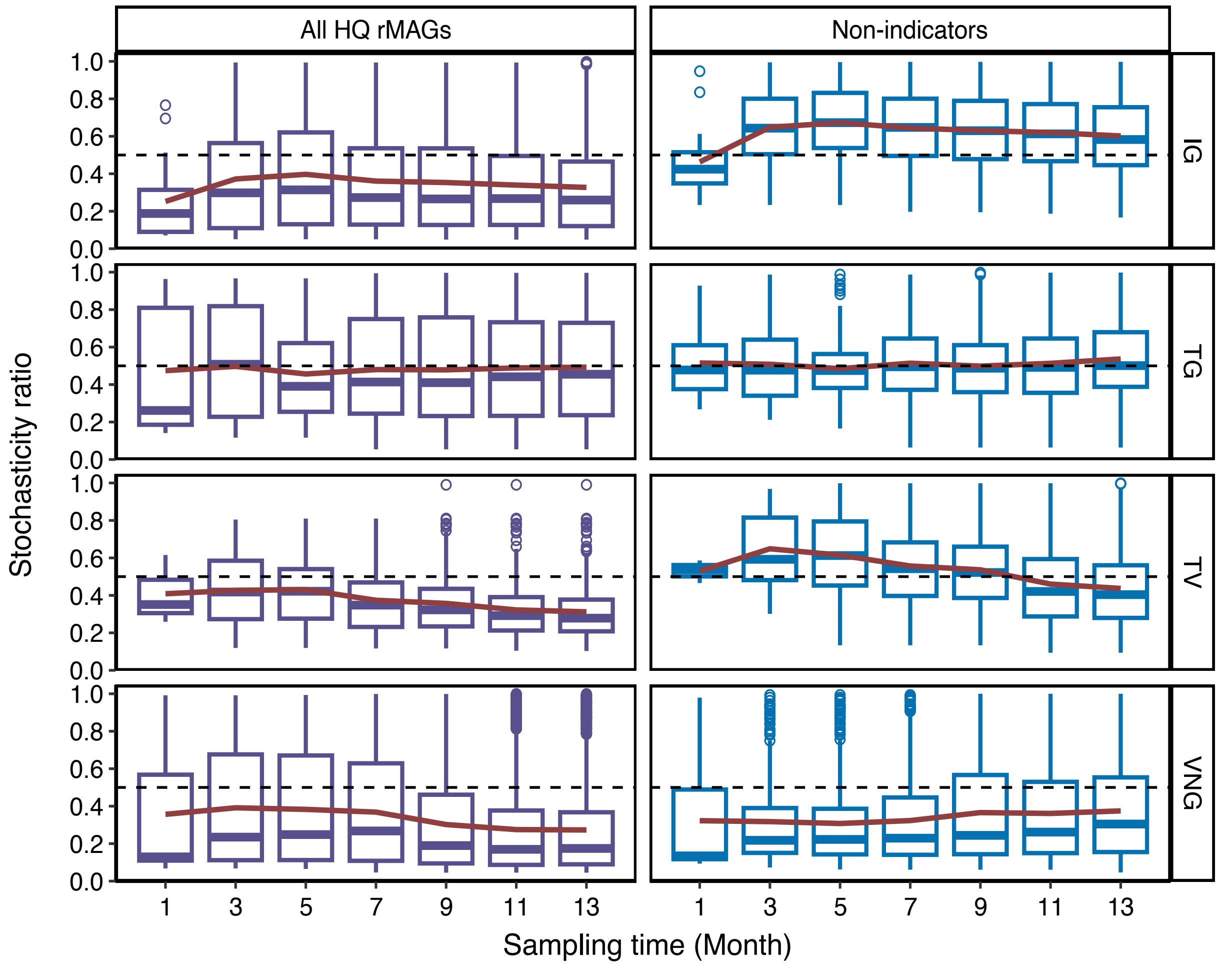

Fig. S11 Stochasticity of the microbial communities of individual plants over time in the (a) activated sludge and (b) anaerobic treatment systems based on all of the high-quality representative metagenome-assembled genomes or non-indicators.

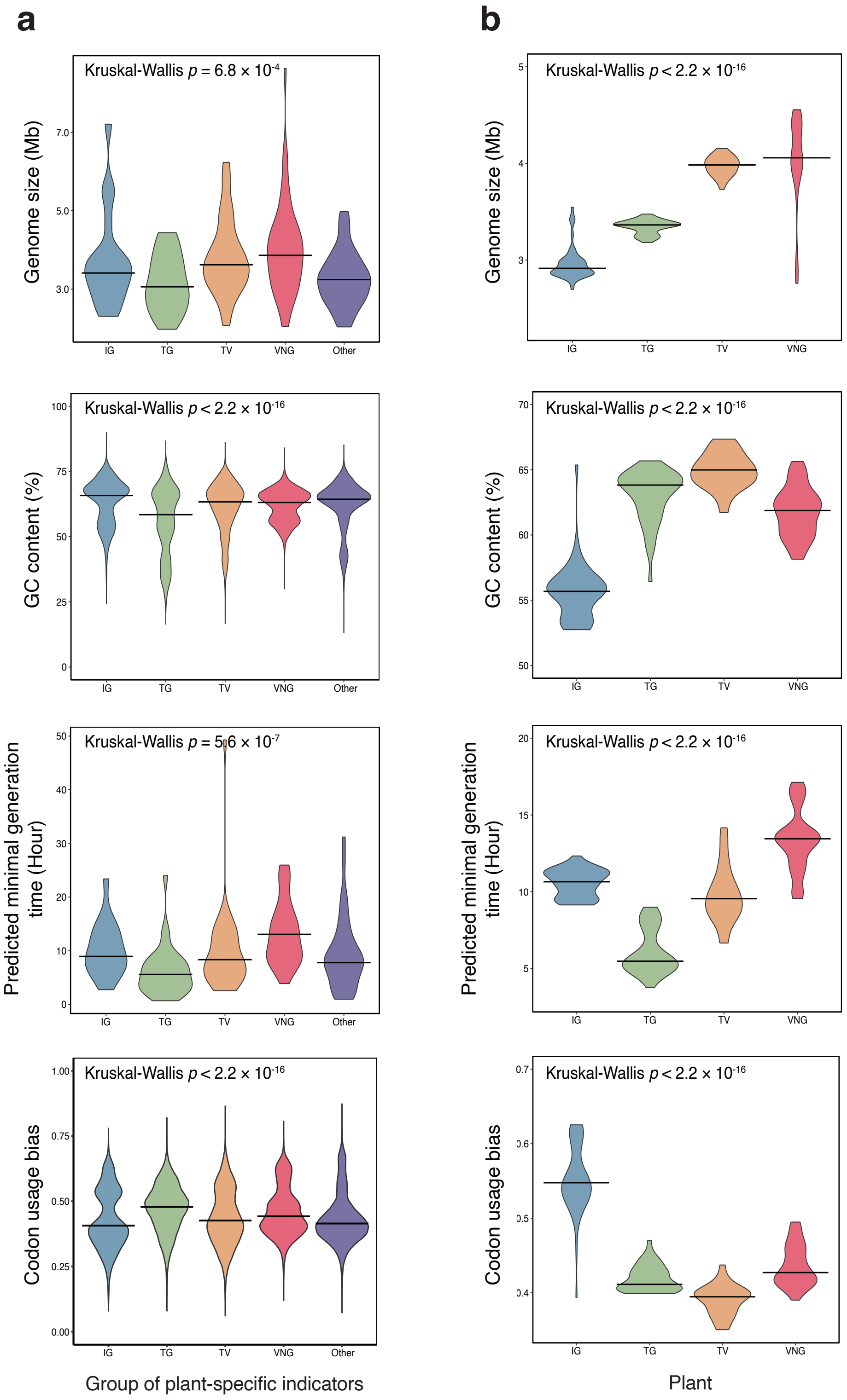

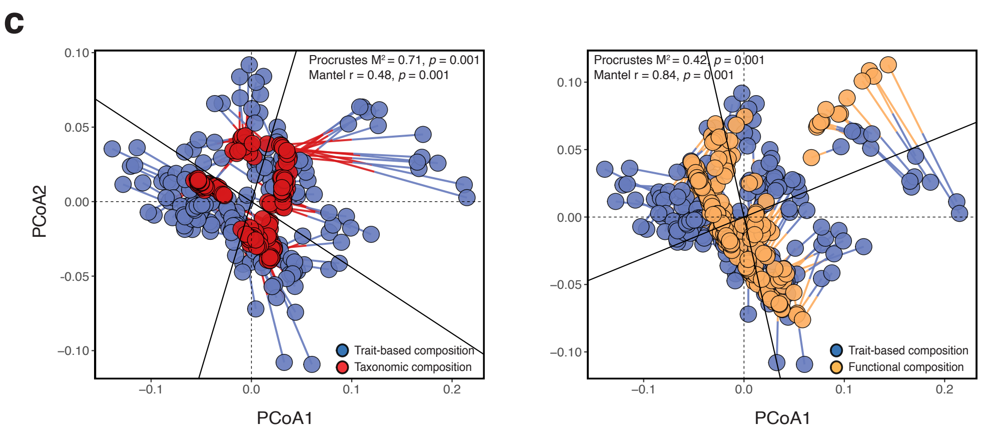

Fig. S12 Genomic traits of microbial communities and similarity between community compositions in the anaerobic treatment systems. (a) Genomic traits of each group of plant-specific indicators. **(b)** Community weighted means of genomic traits of samples from individual plants based on the respective plant-specific indicators. **(c)** Procrustes and Mantel tests of trait-based compositions with taxonomic or functional compositions. The taxonomic, functional, and trait-based composition was calculated based on the HQ rMAGs using the Bray–Curtis dissimilarity.

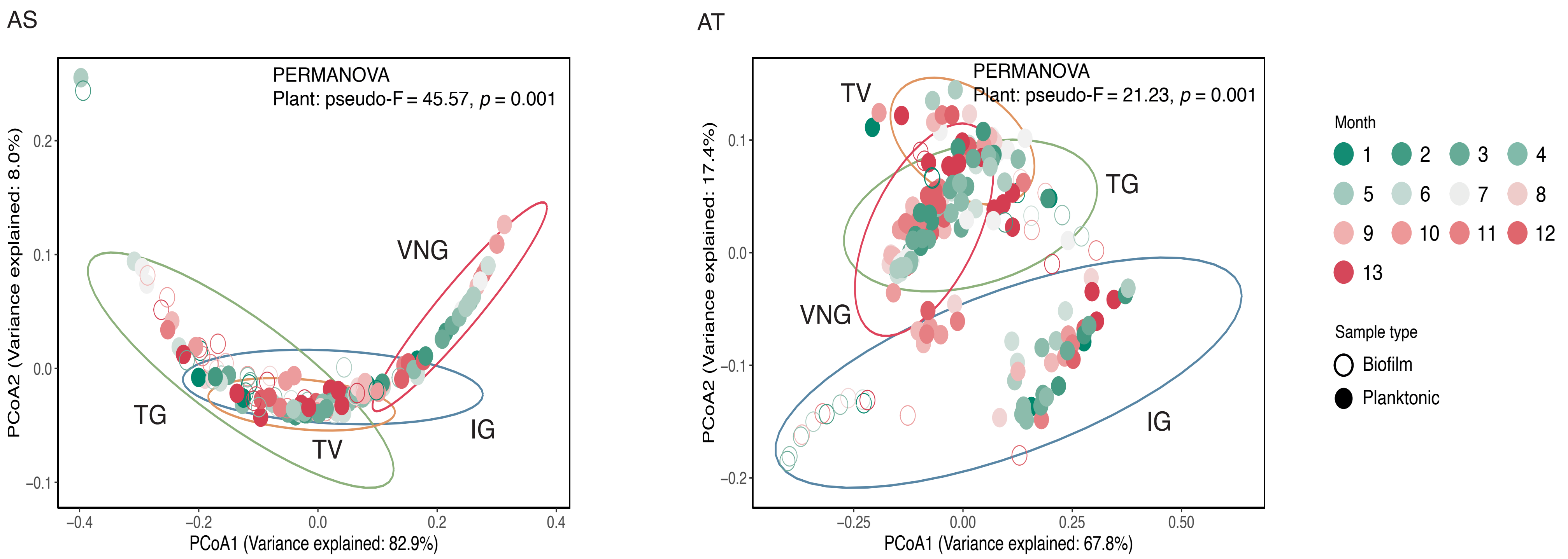

Fig. S13 Principal coordinate analysis of trait-based compositions based on plant-specific indicators in the activated sludge and anaerobic treatment systems. Points are colored according to the month of sampling, and the filled symbols and open symbols indicate planktonic and biofilm samples, respectively. Ellipses are colored based on the multivariate normal distribution at a 95% confidence interval for each plant.

a

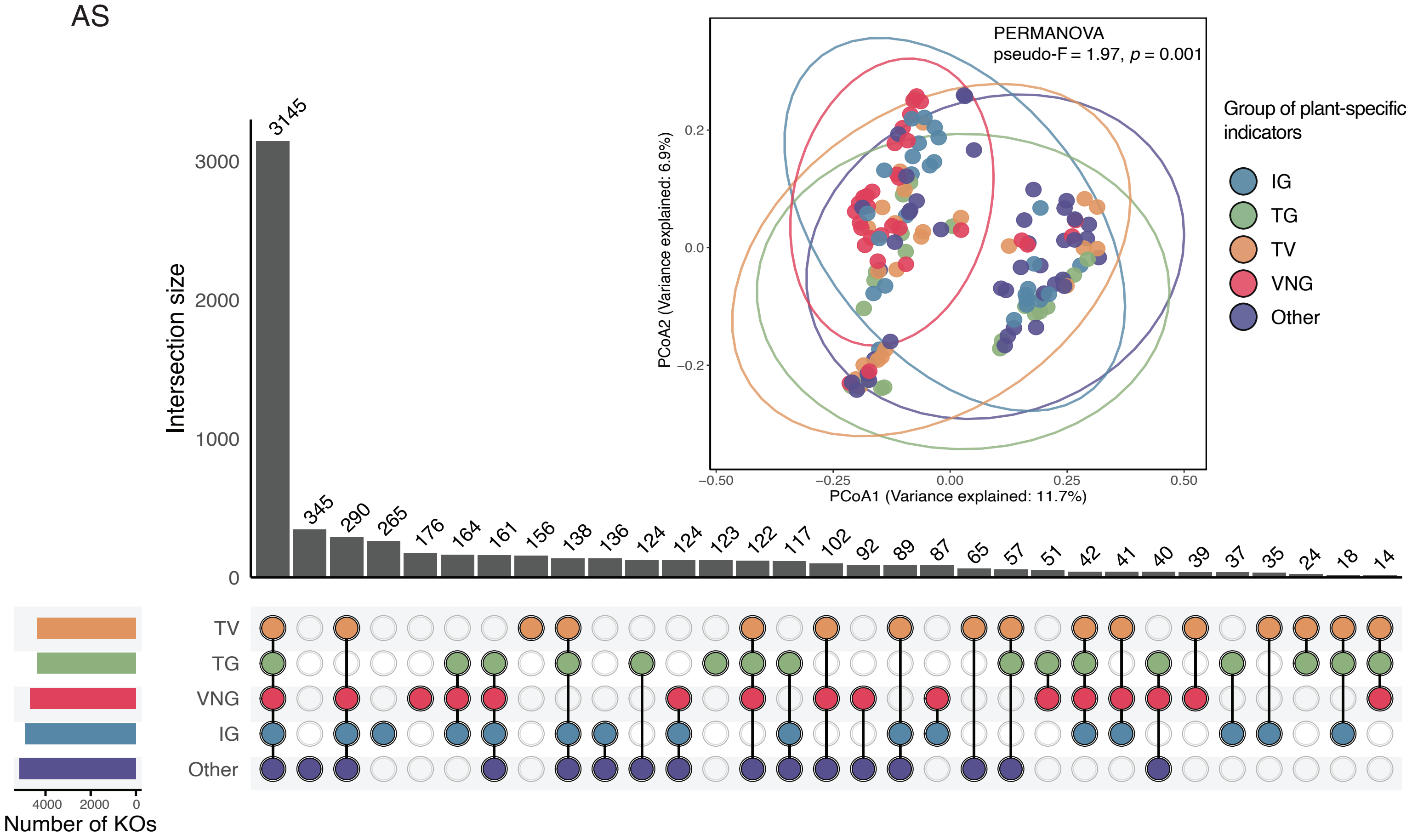
AS
Intersection size
3000
2000
1000
0
3145
345
290
265
176
164
161
156
138
136
124
124
123
122
117
102
92
89
87
65
57
51
42
41
40
39
37
35
24
18
14
TV
TG
VNG
IG
Other
4000
2000
0
Number of KOs
PERMANOVA
pseudo-F = 1.97, p = 0.001
PCoA2 (Variance explained: 6.9%)
PCoA1 (Variance explained: 11.7%)
0.2
0.0
−0.2
−0.50
−0.25
0.00
0.25
0.50
Group of plant-specific indicators
IG
TG
TV
VNG
Other

b

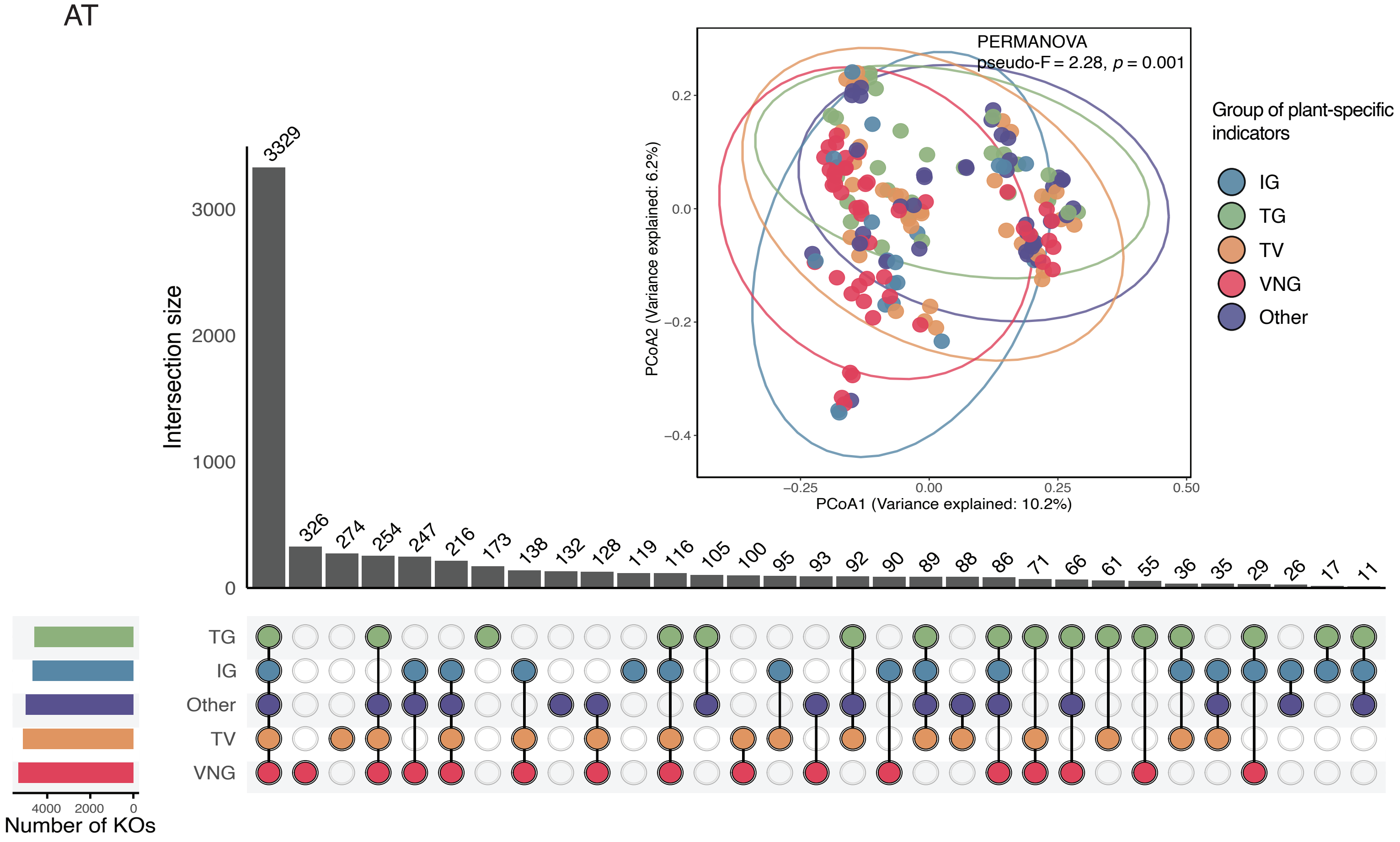
AT
Intersection size
3000
2000
1000
0
3329
326
274
254
247
216
173
138
132
128
119
116
105
100
95
93
92
90
89
88
86
71
66
61
55
36
35
29
26
17
11
TG
IG
Other
TV
VNG
4000
2000
0
Number of KOs
PERMANOVA
pseudo-F = 2.28, p = 0.001
PCoA2 (Variance explained: 6.2%)
PCoA1 (Variance explained: 10.2%)
0.2
0.0
−0.2
−0.4
−0.25
0.00
0.25
0.50
Group of plant-specific indicators
IG
TG
TV
VNG
Other

Fig. S14 Numbers of Kyoto Encyclopedia of Genes and Genomes (KEGG) functional orthologs (KOs) shared between and unique to each group of plant-specific indicators in the (a) activated sludge and (b) anaerobic treatment systems. The colored bars indicate the total number of KOs for each group of plant-specific indicators. The KOs shared by the different groups of plant-specific indicators and the unique KOs of specific groups are represented by the black bars, with the points above black bars indicating the members of the respective groups. The insets show the results of principal coordinate analysis based on the functional repertoire (KOs) of the plant-specific indicators ordinated by the Jaccard distance. Colored ellipses are based on the multivariate normal distribution at a 95% confidence interval for each group of plant-specific indicators.

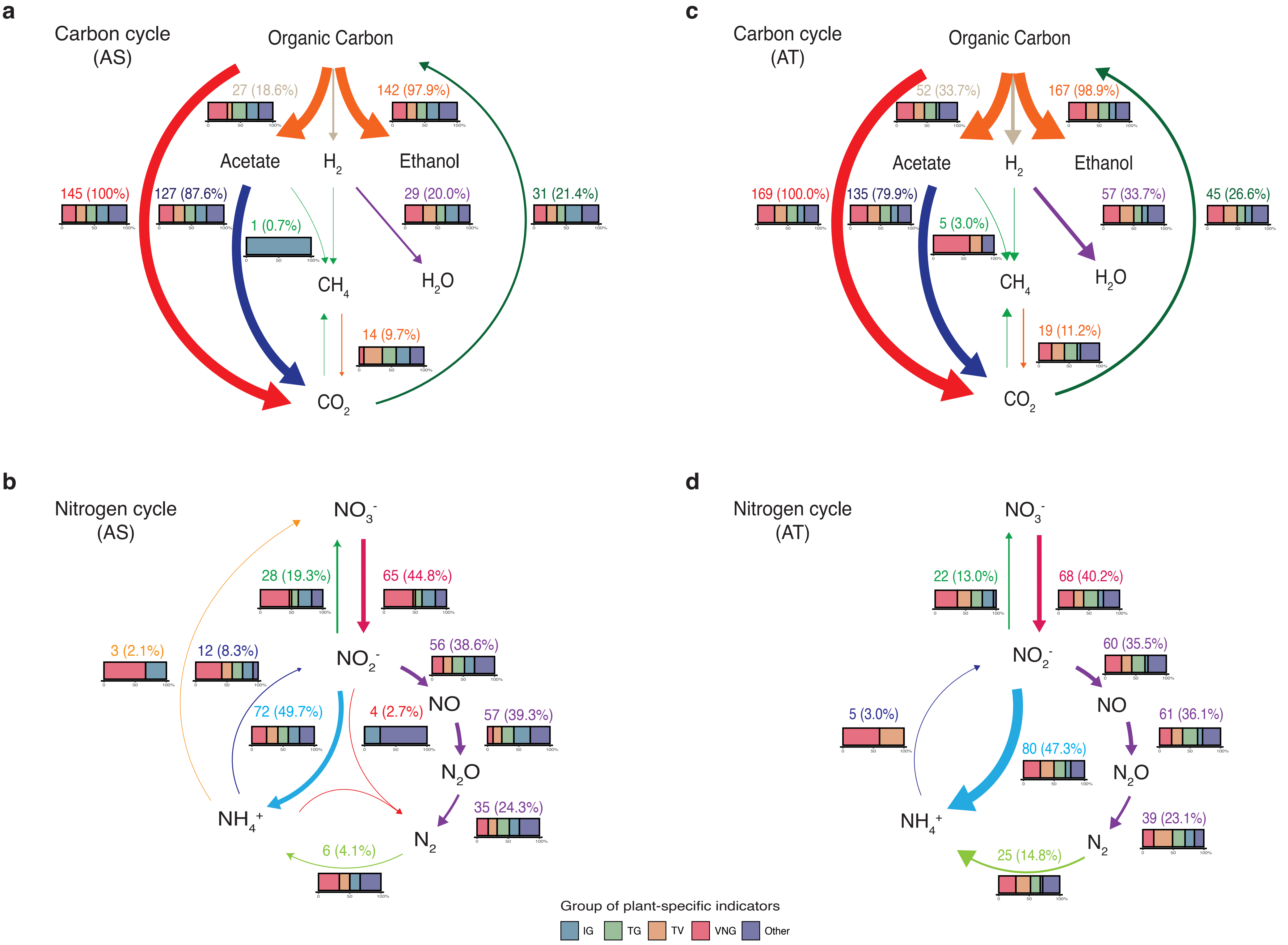
a
Carbon cycle (AS)
Organic Carbon
27 (18.6%)
142 (97.9%)
Acetate
H2
Ethanol
145 (100%)
127 (87.6%)
29 (20.0%)
31 (21.4%)
1 (0.7%)
CH4
H2O
14 (9.7%)
CO2
c
Carbon cycle (AT)
Organic Carbon
52 (33.7%)
167 (98.9%)
Acetate
H2
Ethanol
169 (100.0%)
135 (79.9%)
57 (33.7%)
45 (26.6%)
5 (3.0%)
CH4
H2O
19 (11.2%)
CO2
b
Nitrogen cycle (AS)
NO3-
28 (19.3%)
65 (44.8%)
3 (2.1%)
12 (8.3%)
NO2-
56 (38.6%)
72 (49.7%)
4 (2.7%)
NO
57 (39.3%)
N2O
35 (24.3%)
NH4+
N2
6 (4.1%)
d
Nitrogen cycle (AT)
NO3-
22 (13.0%)
68 (40.2%)
NO2-
60 (35.5%)
5 (3.0%)
NO
61 (36.1%)
80 (47.3%)
N2O
NH4+
39 (23.1%)
N2
25 (14.8%)
Group of plant-specific indicators
IG
TG
TV
VNG
Other

Fig. S15 Potential metabolic functions of the high-quality representative metagenome-assembled genomes (HQ rMAGs) in the activated sludge and anaerobic treatment systems related to (a, c) carbon and (b, d) nitrogen cycles. The thickness of an arrow is proportional to the number of HQ rMAGs capable of carrying out each metabolic step. The numbers of HQ rMAGs and proportions of HQ rMAGs (%) capable of completing each metabolic pathway are indicated. The distribution of the metabolic pathways in each group of plant-specific indicators is shown in a bar plot.

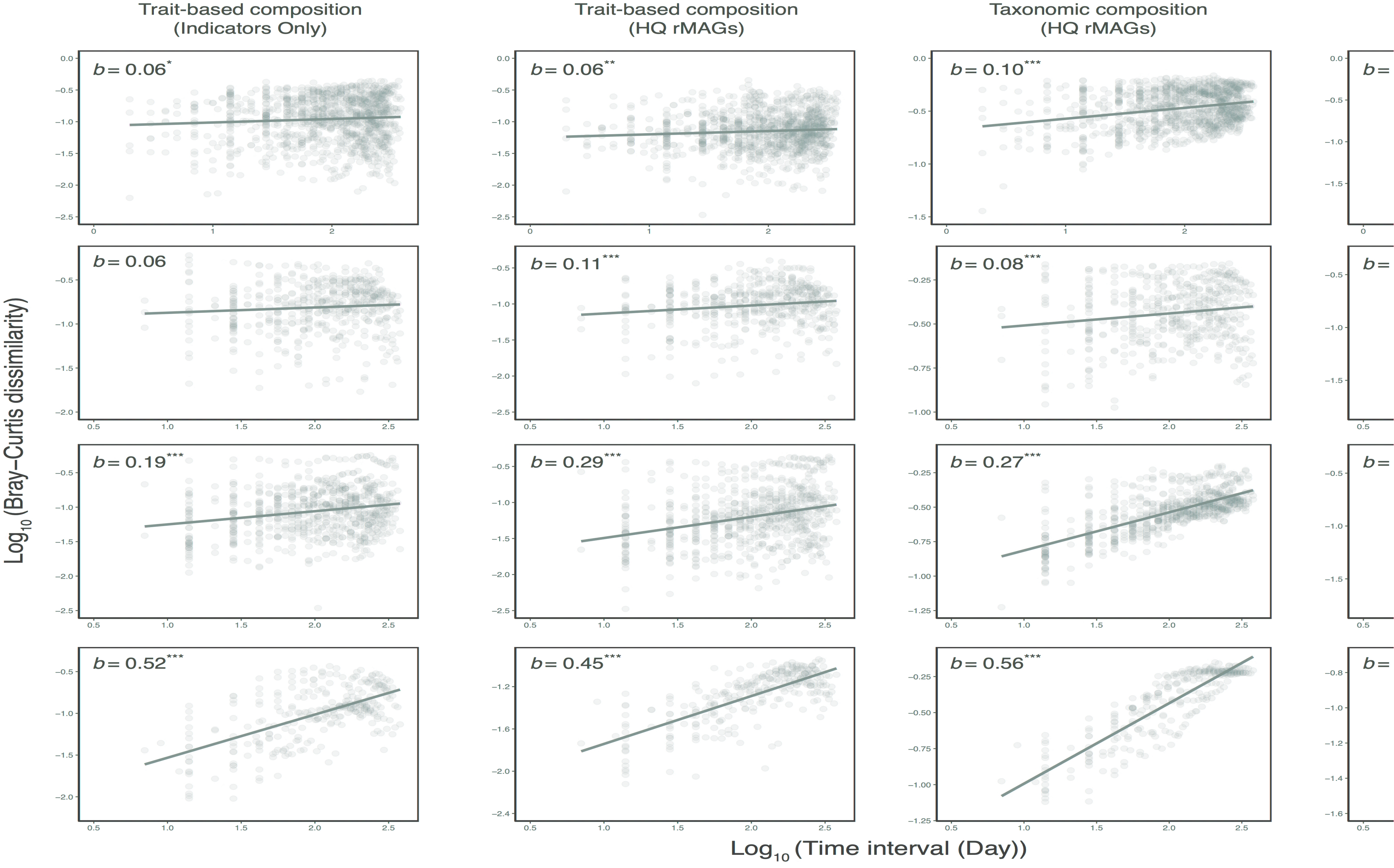

Fig. S16 Time-decay between community dissimilarity and time intervals in the activated sludge systems. The red line represents t between the sample dissimilarity and the time intervals. The strength of the turnover rate was calculated based on the *b* value, which is the (***$p < 0.001$, **$p < 0.01$, *$p < 0.05$). The community dissimilarity for time-decay was calculated based on the Bray–Curtis dissimilarity.

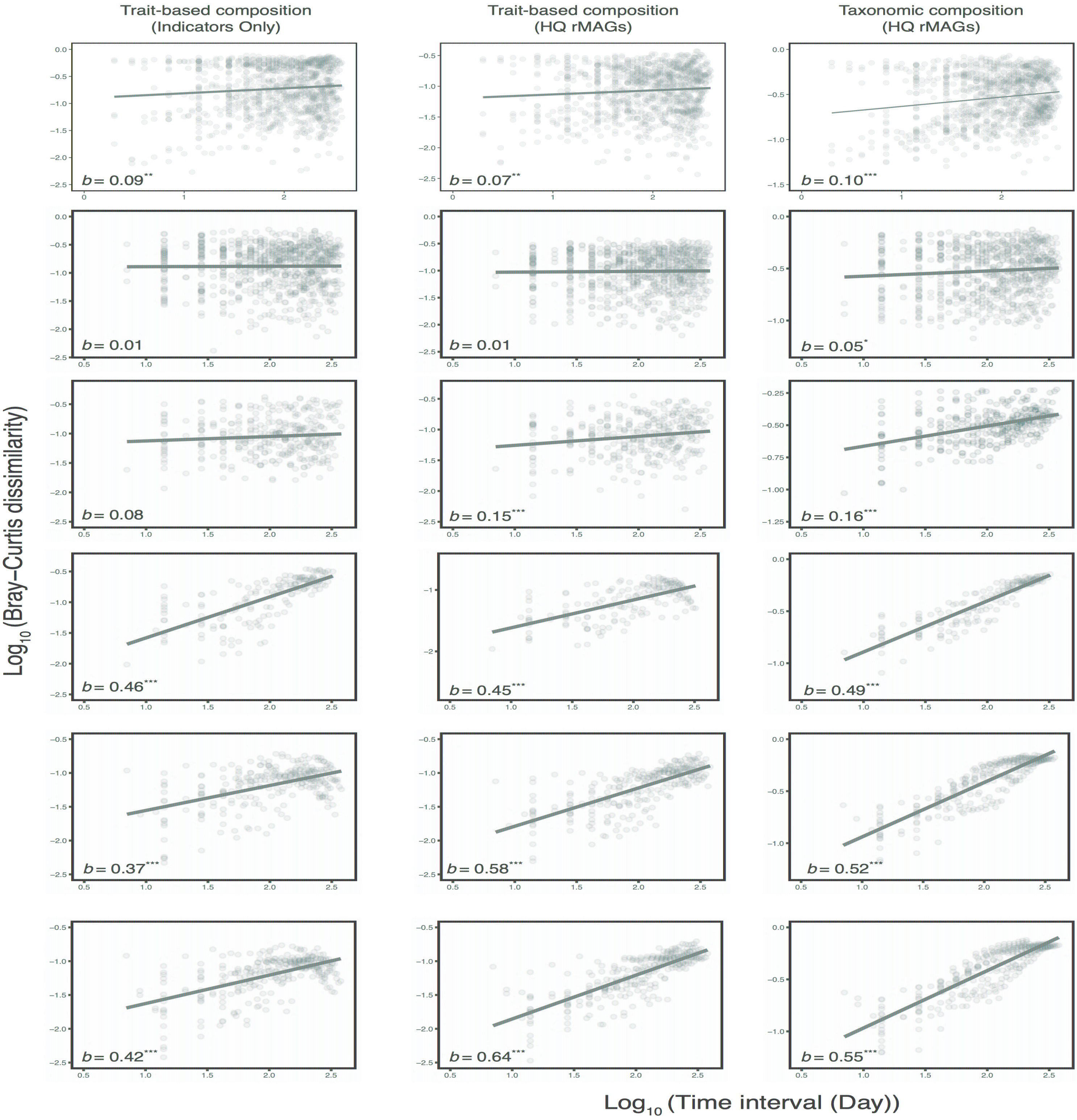

Fig. S17 Time-decay between community dissimilarity and time intervals in the anaerobic line represents the decay of community dissimilarity between the sample dissimilarity and the t the turnover rate was calculated based on the *b* value, which is the slope of the time-decay rel 0.01, **p* < 0.05). The community dissimilarity for time-decay was calculated based on the Bray–

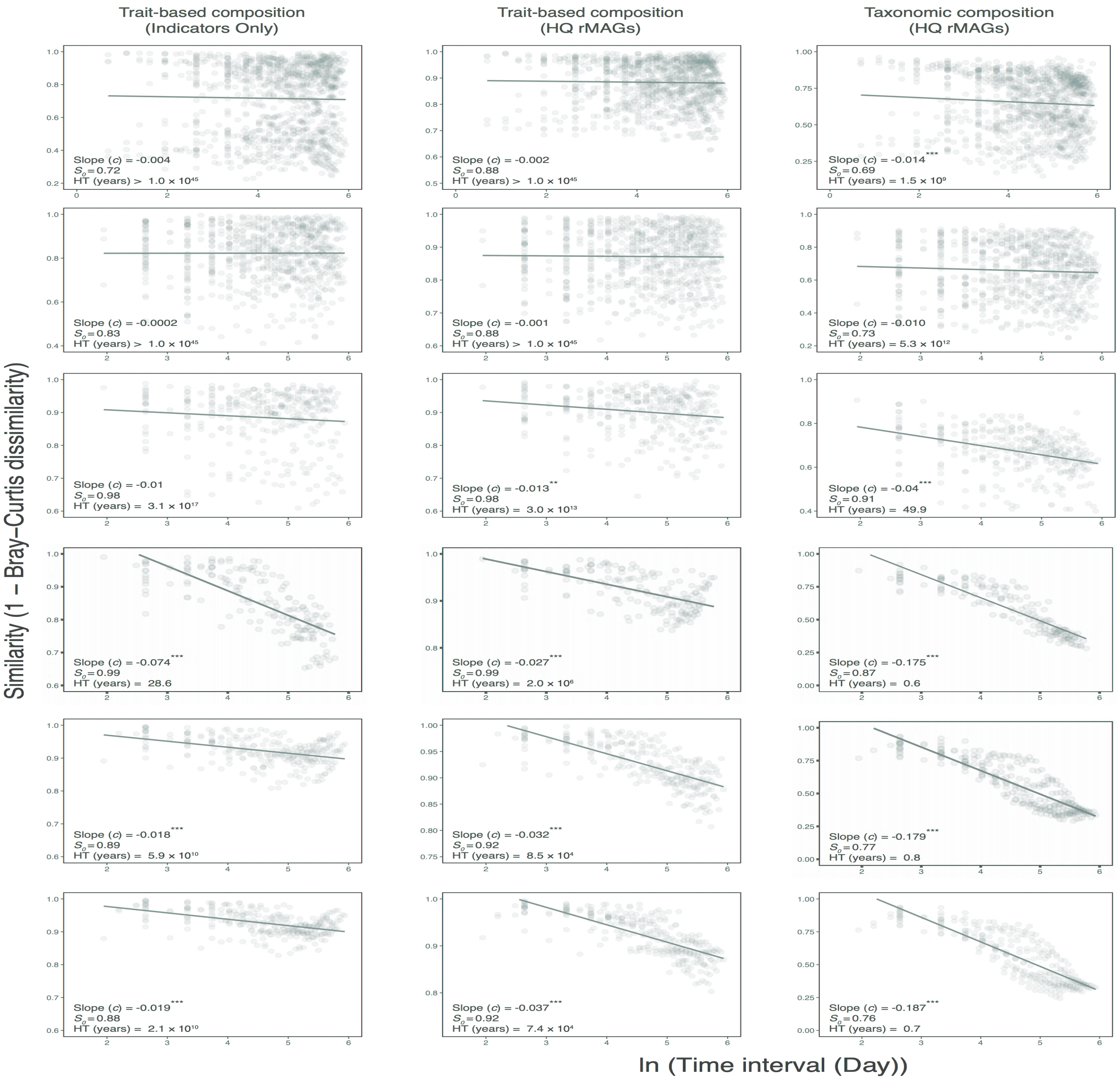

Fig. S18 Halving-time of microbial communities in the anaerobic treatment systems. The of community similarity between the sample similarity and the time intervals. The strength of th calculated using the logarithmic decay model (*** $p < 0.001$, ** $p < 0.01$, * $p < 0.05$). S_o is the initi shortest time interval, and halving-time (HT) is the time required for the community similarity to
